# The causal relationship between autoimmune diseases and rhinosinusitis, and the mediating role of inflammatory proteins: a Mendelian randomization study

**DOI:** 10.3389/ebm.2024.10196

**Published:** 2024-07-22

**Authors:** Yanjing Liang, Shao Yin, Xiangyan Chen, Chengen Li, Qiu Chen

**Affiliations:** ^1^ Chengdu University of Traditional Chinese Medicine, Chengdu, China; ^2^ Hospital of Chengdu University of Traditional Chinese Medicine, Chengdu, China

**Keywords:** autoimmune diseases, rhinosinusitis, inflammatory factors, mediating role, Mendelian randomization

## Abstract

Observational studies have linked autoimmune diseases (ADs) with rhinosinusitis (RS) manifestations. To establish a causal relationship between ADs and RS, and to explore the potential mediating role of inflammatory mediators between ADs and RS, we utilized Mendelian randomization (MR) analysis. Using a two-sample MR methodology, we examined the causality between multiple sclerosis (MS), rheumatoid arthritis (RA), ankylosing spondylitis (AS), psoriasis (PsO), type 1 diabetes (T1D), Sjogren’s syndrome (SS), celiac disease (CeD), Crohn’s disease (CD), hypothyroidism (HT), Graves’ disease (GD), and Hashimoto’s thyroiditis and their association with chronic and acute rhinosinusitis (CRS and ARS, respectively).To achieve this, we employed three distinct MR techniques: inverse variance weighting (IVW), MR-Egger, and the weighted median method. Our analysis also included a variety of sensitivity assessments, such as Cochran’s Q test, leave-one-out analysis, MR-Egger intercept, and MR-PRESSO, to ensure the robustness of our findings. Additionally, the study explored the role of inflammation proteins as a mediator in these relationships through a comprehensive two-step MR analysis. Among the ADs, MS, RA, T1D, CeD, and HT were determined as risk factors for CRS. Only CeD exhibited a causal relationship with ARS. Subsequent analyses identified interleukin-10 (IL-10) as a potential mediator for the association of MS, RA and HT with CRS, respectively., while C-X-C motif chemokine 10 levels (CXCL10) and T-cell surface glycoprotein CD6 isoform levels (CD6) were found to influence HT’s effect on CRS. Our findings demonstrate a causative link between specific autoimmune diseases and rhinosinusitis, highlighting IL-10, CXCL10, and CD6 as potential mediators in this association.

## Impact statement

This study establishes a causal link between several autoimmune diseases (ADs)—specifically multiple sclerosis, rheumatoid arthritis, type 1 diabetes, celiac disease, and hypothyroidism—and rhinosinusitis, employing Mendelian randomization (MR) techniques, including inverse variance weighting, MR-Egger, and weighted median methods, alongside a variety of sensitivity assessments. By demonstrating that several ADs are risk factors for chronic rhinosinusitis and identifying celiac disease as a causal factor for acute rhinosinusitis, this work advances understanding of the interplay between autoimmune disorders and sinusitis. Crucially, it identifies inflammatory mediators such as interleukin-10, C-X-C motif chemokine 10, and CD6 isoform levels as key components in these relationships, offering new insights into potential therapeutic targets. These findings significantly impact the field by providing updated clues of the pathological links between autoimmune conditions and sinus health, with implications for treatment strategies.

## Introduction

Autoimmune diseases (ADs), characterized by the immune system’s disarray leading to self-directed tissue and organ damage, include diseases like multiple sclerosis (MS), rheumatoid arthritis (RA), type 1 diabetes (T1D), systemic lupus erythematosus (SLE), celiac disease (CeD), Crohn’s Disease (CD), and autoimmune thyroid diseases (AITD). Their development is tightly linked to genetics, environmental influences, and shifts in the microbiota [1–3]. Epidemiologically, the escalating incidence of ADs is recognized as a significant global health menace, posing a formidable challenge to public health systems [4, 5]. While treatment approaches have evolved, there’s a continuing need for tailored treatment plans and profound investigations into the mechanisms of these complex diseases.

Divided into acute and chronic categories, rhinosinusitis (RS) adversely affects the sinus mucous membranes [6]. The acute rhinosinusitis (ARS) presents with symptoms such as nasal congestion, secretion of mucoid or purulent material, facial discomfort, and olfactory reduction, not exceeding 4 weeks in duration [6, 7]. Chronic rhinosinusitis (CRS), lasting over 12 weeks, invariably involves nasal inflammation, often with preceding rhinitis symptoms [6, 8]. Its occurrence is tightly linked with factors including bacterial, viral, genetic, immune deficiency, environmental components, and air quality [8, 9], with nasal polyps and turbinate enlargement also contributing to the severity [10, 11]. Management options range from antibiotics, vasoconstrictors, antihistamines, and hypertonic saline for symptomatic relief to surgical procedures, with treatment efficacy potentially influenced by factors such as weather and emotional states [9, 12].

Inflammation is a key player in the progression of diseases, holding significant importance for both science and public health. Triggered by bacteria such as *Streptococcus* pneumoniae and viruses like coronaviruses, ARS involves the activation of various inflammatory cytokines, including IFN-α, IL-1β, and IL-6 [13, 14]. CRS, mirroring the complexity of chronic diseases, poses a significant challenge in finding a cure. The expression and regulation of cytokines are paramount in its etiology. CRS inflammation is differentiated into three categories based on the elevation of specific lymphocyte cytokines: Types 1, 2, and 3 [15, 16]. The complexity of interactions between immune cells and cytokines plays a critical role in CRS-related inflammation and tissue remodeling [17]. As mediators of communication among immune cells, cytokines are crucial in the regulation of immune responses and inflammation [18]. Aberrant cytokine levels in autoimmune diseases can result in sustained inflammation, leading to tissue damage and advancement of the disease [18, 19]. It has been indicated that autoimmune diseases could heighten the risk of CRS, influencing its prognosis [20, 21]. While the direct causal link remains to be established, the association between immune dysfunctions and inflammatory responses directs future research towards understanding the interplay between these conditions and enhancing therapeutic approaches.

Mendelian randomization (MR) utilizes genetic variants as instruments to investigate the causal relationships between particular exposures and outcomes, leveraging single nucleotide polymorphisms (SNPs) from genome-wide association studies (GWAS) to bypass traditional study confounders and enhance causal inference accuracy. This study is centered on exploring the Mendelian causality between ADs and RS, specifically focusing on the role of inflammatory mediators. Employing large GWAS datasets from European populations, through dual-sample MR analysis and a two-phase mediation approach, our aim is to reveal both the direct and indirect causal links between ADs and RS, contributing to a deeper biological understanding of their mutual influences.

## Materials and methods

### Study design

**FIGURE 1:**
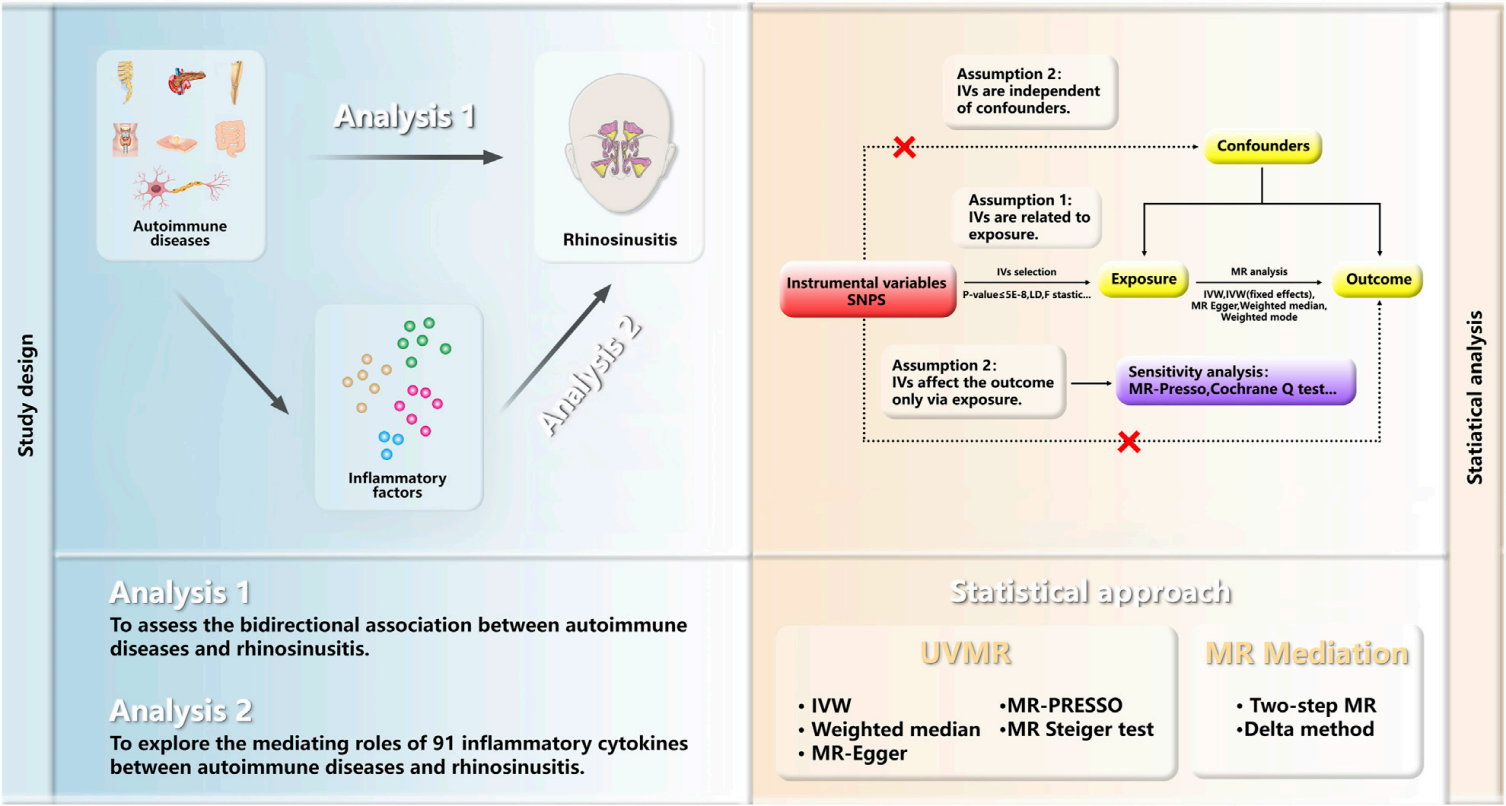
Overview of the MR Study Design. The study is structured into two analytical phases. Analysis 1 employed UVMR to investigate the causal links between ADs and RS. Analysis 2 utilized a two-step MR approach to explore the potential mediation effect of inflammatory proteins on the causal association between ADs and RS. MR, Mendelian randomization; UVMR, univariable Mendelian randomization analyses; IVs, Instrumental variables; SNPs, single-nucleotide polymorphisms; LD, linkage disequilibrium; WM, weighted median; IVW, inverse variance weighted; PRESSO pleiotropy residual sum and outlier.

To delineate the causal dynamics and possible mediating roles between ADs and RS, chronic and acute, this study embarked on a two-phased Mendelian randomization approach. The initial phase employed univariable MR (UVMR) to explore the causal relationships between ADs and RS. Only exposures with a significant causal link to RS, and where reverse causation was excluded, were subjected to further scrutiny. The subsequent phase was dedicated to identifying and measuring the influence of circulating inflammatory proteins as mediators in the causal pathway from ADs to RS. Conducted in accordance with the “Strengthening the Reporting of Observational Studies in Epidemiology Using Mendelian Randomization,” this investigation required no additional ethical approvals or informed consent, leveraging publicly accessible GWAS data. The design and analytical procedure are summarized in Figure 1 (All references in the Methods section are listed in Supplementary Table S1).

### Data sources

Employing publicly accessible GWAS summary datasets, this MR study assessed designated traits (refer to Table 1). For T1D, genetic tools were sourced from a meta-analysis involving UK and Sardinian participants (7,467 cases versus 10,218 controls). The RA dataset originated from a meta-analysis involving 14,361 cases and 43,923 controls, while genetic instrument variables (IVs) for MS were furnished by a German study (4,888 cases and 10,395 controls). Data for Ankylosing spondylitis (AS) were contributed by the International Genetics of Ankylosing Spondylitis Consortium (IGAS) (9,069 cases and 13,578 controls), and IVs for Psoriasis (PsO) came from research involving 15,967 cases and 28,194 controls. Systemic lupus erythematosus (SLE) data involved 5,201 European descent cases and 9,066 controls. The IEU GWAS database provided information for Sjogren’s syndrome (407,746 samples), Celiac disease (11,812 cases and 11,837 controls), Hypothyroidism or myxoedema (405,357 samples), and Crohn’s disease (17,897 cases and 33,977 controls). Graves’ disease (1,678 cases and 456,942 controls) and Hashimoto thyroiditis (15,654 cases and 379,986 controls) data were derived from a meta-analysis of the European population.

**TABLE 1:** Data source details for our GWAS study.

	Phenotype	Data source	Cases	Control	Sample size	Ancestry	PIMD
Exposures	Type 1 diabetes	Inshaw JRJ et al.	7,467	10,218	17,685	European	33830302
	Rheumatoid arthritis	Ha E et al.	14,361	43,923	58,284	European	33,310,728
	Multiple sclerosis	Andlauer TF et al.	4,888	10,395	15,283	European	27,386,562
	Ankylosing spondylitis	Cortes A et al.	9,069	13,578	22,647	European	23,749,187
	Psoriasis	Stuart PE et al.	15,967	28,194	44,161	European	34,927,100
	Systemic lupus erythematosus	Bentham J et al.	5,201	9,066	14,267	European	26,502,338
	Sjogren’s syndrome (SPA correction)	Mbatchou J et al.	NA	NA	407,746	European	34,017,140
	Celiac disease	Trynka G et al.	11,812	11,837	23,649	European	22,057,235
	Crohn’s disease	Liu JZ et al.	17,897	33,977	51,874	European	26,192,919
	Hypothyroidism or myxoedema (SPA correction)	Mbatchou J et al.	NA	NA	405,357	European	34,017,140
	Graves' disease	Sakaue S et al.	1,678	456,942	458,620	European	34,594,039
	Hashimoto thyroiditis	Sakaue S et al.	15,654	379,986	395,640	European	34,594,039
outcome	Acute sinusitis	Finngen	10,916	182,945	193,861	European	NA
	Chronic sinusitis	Finngen	8,524	167,849	176,373	European	NA
Mediator	Circulating inflammatory proteins	Zhao JH et al.	NA	NA	14,824	European	26,192,919

Data regarding rhinosinusitis cases were retrieved from the FinnGen project’s R10 dataset, involving 412,181 individuals with European descent, documenting 21,311,942 variants. Summary GWAS results for ARS (with 10,916 cases against 182,945 controls) and CRS (8,524 cases compared to 167,849 controls) were acquired from FinnGen^1^. Information on circulating inflammatory proteins, considered as potential mediators, was taken from a recent European study spanning 11 cohorts, which analyzed 91 plasma proteins across 14,824 samples and performed a comprehensive genome-wide protein quantitative trait loci (pQTL) study. Detailed data on the inflammatory proteins can be found in Supplementary Table S2.

### Selection criteria for IVs

When selecting appropriate genetic IVs from GWAS of 12 ADs using the TwoSampleMR package in R studio (default settings), we primarily chose SNPs with *p*-values less than 5.0E-08 to ensure genome-wide significance. Specifically, for Sjogren’s syndrome (SS), due to a lack of SNPs meeting the *p* < 5.0E-08 criterion, we lowered the threshold to *p* < 5.0E-06 to secure a sufficient number of valid IVs. MR-Steiger analysis was utilized to assess the direction and validity of causal relationships, with *p* > 0.05 indicating potential reverse causation, prompting reverse MR analysis. Lower significance threshold to consider *p* < 5.0E-06 was also adopted when analyzing the potential mediating effects of inflammatory proteins. Independence of SNPs was ensured using an *r* ^2^ = 0.001 and a clumping window of 10,000 kb. The validity of all IVs was evaluated based on the F-statistic (F = β^2/se^2), considering IVs effective when F values exceeded 10.

### Statistical analysis

#### Univariable Mendelian randomization

A UVMR approach was used to examine causal links between ADs and RS, including inverse-variance weighted (IVW), weighted median, and MR-Egger analysis. The IVW method, serving as the main approach, offers precise causal estimates under the condition that all IVs are valid. MR-Egger method, which offers adjustments for estimate heterogeneity and an intercept term for evaluating pleiotropy, enhances the robustness of causal inference. The weighted median approach, effective when a majority of SNPs are credible IVs, allows for an unbiased estimation of causality. Additionally, MR-Steiger analysis was utilized to verify causal direction and mitigate reverse causality concerns among SNPs.

#### Mediation analysis

After conducting UVMR to assess causal links between ADs and RS, significant causations were selected for mediation effect analysis in a two-step approach. The research delved into the potential mediating roles of 91 inflammatory proteins between ADs and RS. The total causal effect of ADs on RS (β) was first identified via UVMR, followed by an evaluation of the causal influences of ADs on 91 inflammatory proteins (β1) and their subsequent effects on RS (β2). Diseases with evidence of reverse causality were excluded from the mediation analysis. The correlation between inflammatory proteins and the risk for ADs or RS was assessed via two step MR analyses, using the IVW method with *p* < 0.05 indicating statistical significance. For inflammatory proteins related to both ADs and RS, the mediation effect was examined, calculated as the product of β1 and β2 divided by β, and the proportion of mediation effect was estimated using the delta method for 95% confidence intervals (CI).

#### Sensitivity analysis

This research applied MR-Egger and weighted median methods for sensitivity analysis to confirm the UVMR analysis’s robustness, additionally utilizing the Cochran Q test to examine IVs heterogeneity. The MR-Egger intercept was deployed to evaluate pleiotropy, depicted in scatter diagrams. High-heterogeneity SNPs were removed through a leave-one-out procedure, with a subsequent reassessment of outcomes. MR-Presso identified and eliminated outlier SNPs, enhancing estimation accuracy. MR-Steiger testing further affirmed causal direction and reliability. The analyses utilized R software (version 4.0.2), specifically through the “TwoSampleMR” (version 0.5.8), “MendelianRandomization” (version 0.9.0), and “MRPRESSO” packages, as detailed in Supplementary Table S1.

## Results

### Effects of ADs on RS

**FIGURE 2:**
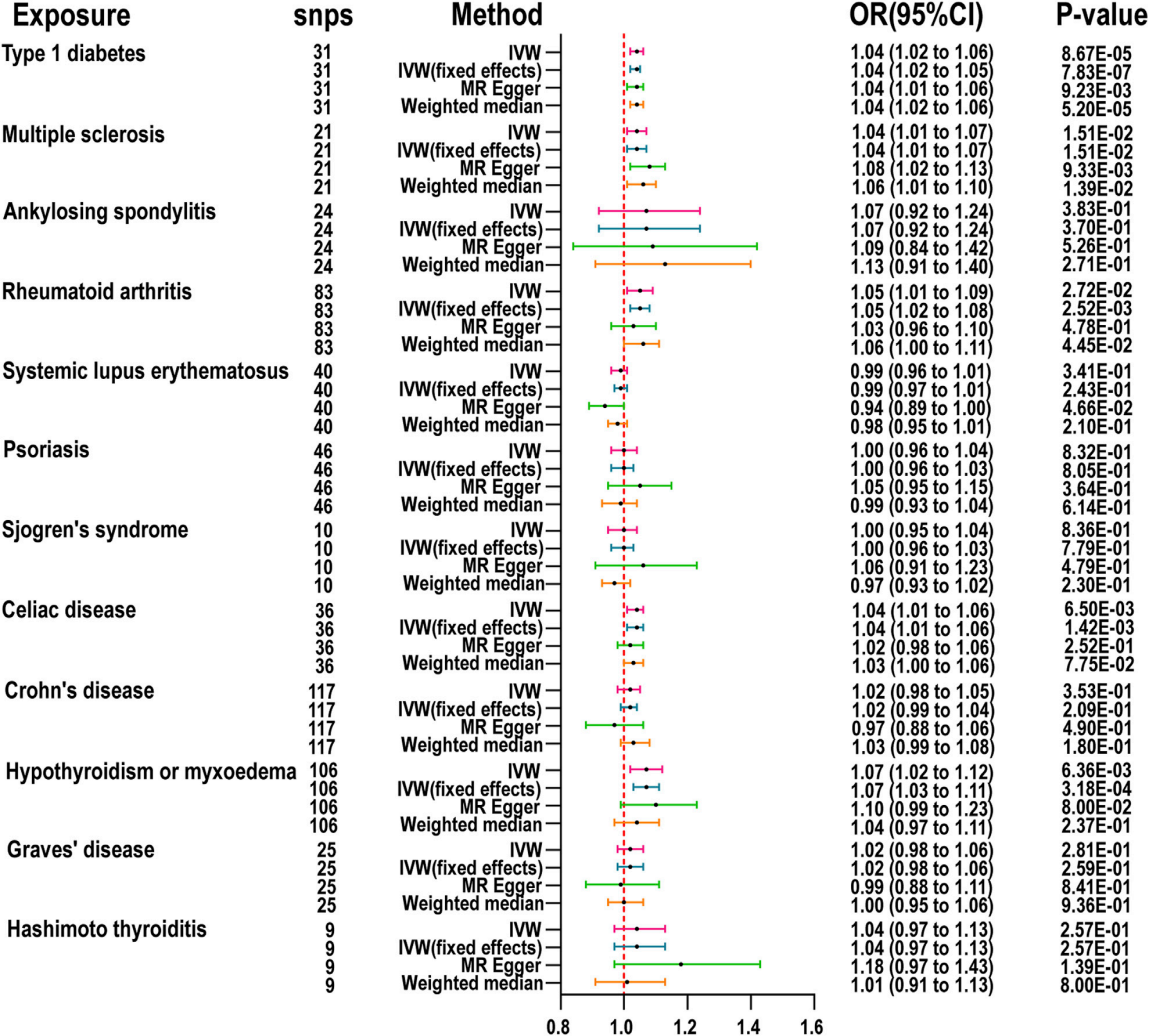
MR is used to examine the link between 12 autoimmune diseases and chronic rhinosinusitis. inverse variance weighted (IVW), number of SNPs used in MR (nsnps), odds ratio (OR), confidence interval (Cl).

The MR results for the impact of ADs on CRS are detailed in Figure 2. After rigorous selection and exclusion, the IVs demonstrated F-statistics greater than 10, indicating no weak instrument bias (Supplementary Table S3). If significant heterogeneity was detected (Cochrane Q test *p*-value <0.05), the random effects model of the IVW method was employed as the primary analytical approach; otherwise, the fixed effects model of IVW was utilized. Two-sample MR (TSMR) revealed that the genetic predisposition to CRS is significantly associated with MS (OR = 1.04, 95% CI: 1.01-1.07, *p =* 1.51E-02), CeD (OR = 1.04, 95% CI: 1.01-1.06, *p =* 1.42E-03), RA (OR = 1.05, 95% CI: 1.01-1.09, *p =* 2.72E-02), T1D (OR = 1.04, 95% CI: 1.02-1.06, *p =* 8.67E-05), and hypothyroidism or myxedema (OR = 1.07, 95% CI: 1.02-1.12, *p =* 6.36E-03). The MR-Egger intercept analysis did not demonstrate horizontal pleiotropy, and MR-Steiger testing validated the directionality of our causal inferences (Supplementary Table S4). Scatter plots, funnel plots, and leave-one-out analyses further describe the results (Supplementary Figures S1–12). Similarly, using two-sample MR and the IVW method, only the relationship between CeD and ARS was significant (OR = 1.04, 95% CI: 1.01-1.06, *p =* 2.07E-03). The MR results for ADs on ARS are presented in Supplementary Figure S13, with sensitivity analyses and MR-Steiger test results in Supplementary Table S4. Scatter plots, funnel plots, and leave-one-out analyses further detail these findings (Supplementary Figures S14–25).

### Mediating effect of inflammatory proteins

**FIGURE 3:**
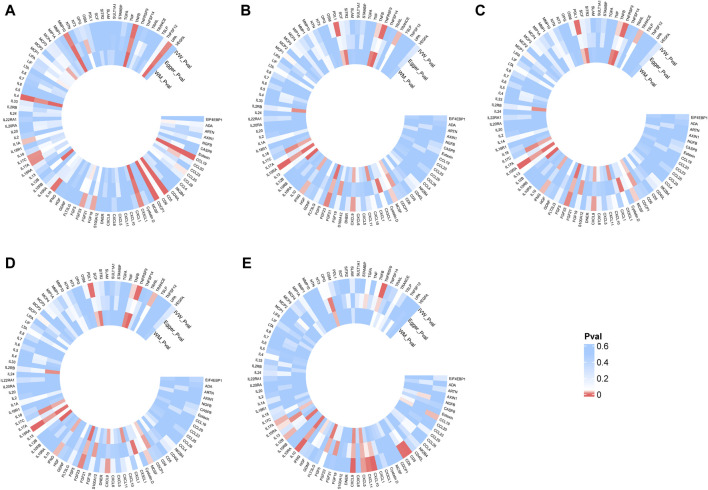
Heatmap of the causal relationships between five autoimmune diseases and 91 inflammatory proteins. **(A)**: Effects of multiple sclerosis on inflammatory proteins. **(B)**: Effects of celiac disease on inflammatory proteins. **(C)**: Effects of type 1 diabetes on inflammatory proteins; **(D)**, Effects of rheumatoid arthritis on inflammatory proteins. **(E)**: Effects of hypothyroidism or myxedema on inflammatory proteins. The color gradient from blue to red indicates an increasing significance of the causal relationship.inverse variance weighted (IVW), number of SNPs used in MR (nsnps), odds ratio (OR), confidence interval (Cl).

**FIGURE 4:**
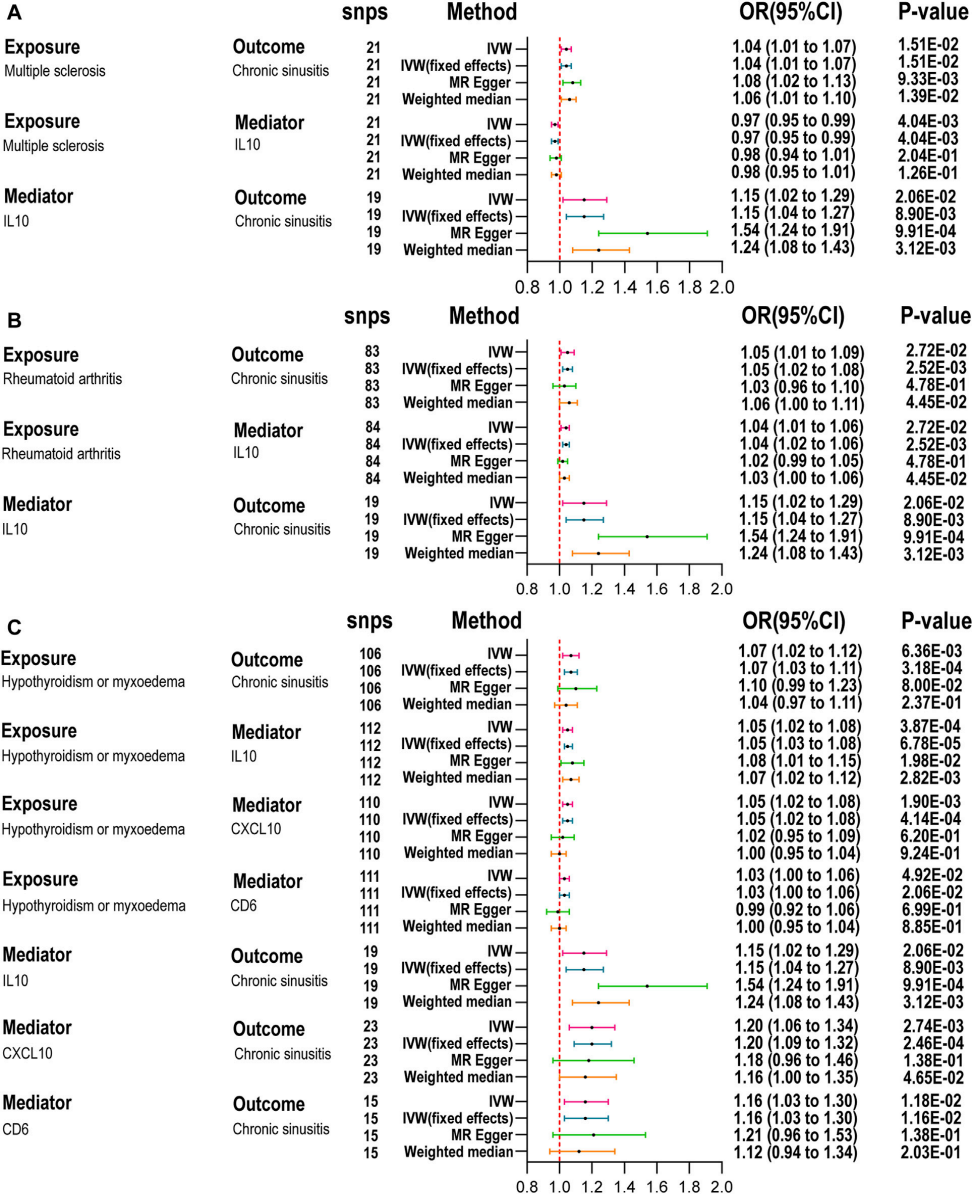
Mediation analysis of the effect of autoimmune diseases on chronic rhinosinusitis via inflammatory proteins. **(A)**: Summary MR for the effect of MS on CRS via inflammatory factor. **(B)**: Summary MR for the effect of RA on CRS via inflammatory factor. **(C)**: Summary MR for the effect of HT on CRS via inflammatory factor; inverse variance weighted (IVW), number of SNPs used in MR (nsnps), odds ratio (OR), confidence interval (Cl) Table 2. IL-10, nterleukin-10; CXCL10, C-X-C motif chemokine 10; CD6, T-cell surface glycoprotein CD6 isoform.

Following the MR analysis between ADs and RS, we identified five ADs with a close relationship to RS and compared them against 91 circulating inflammatory proteins. Analysis using TSMR and the IVW method revealed that 15, 10, 9, 7, and 12 inflammatory proteins were significantly associated with MS, CeD, RA, T1D, and hypothyroidism (HT), respectively (Figure 3). To investigate which specific inflammatory proteins could serve as potential mediators in the causal relationship between ADs and RS, we analyzed those proteins significantly associated with ADs as new exposures in relation to RS. The MR-Steiger test was employed to validate the direction of causality, excluding reverse causal associations from mediation analysis. After SNP filtering and MR analysis, only inflammatory proteins significantly related to both ADs and RS were considered for mediation analysis. The IVW method indicated that interleukin-10 levels (IL-10) were significantly associated with MS (OR = 0.97, 95% CI: 0.95–0.99, *p =* 4.04E-03), RA (OR = 1.04, 95% CI: 1.01–1.06, *p =* 1.13E-03), and HT (OR = 1.05, 95% CI: 1.02–1.08, *p =* 3.87E-04), as detailed in Figure 4. Additionally, T-cell surface glycoprotein CD6 isoform levels (CD6) were significantly related to HT (OR = 1.03, 95% CI: 1.00–1.06, *p =* 4.92E-02) and CRS (OR = 1.16, 95% CI: 1.03–1.30, *p =* 1.16E-02), and C-X-C motif chemokine 10 levels (CXCL10) to HT (OR = 1.05, 95% CI: 1.02–1.08, *p =* 1.90E-03) and CRS (OR = 1.20, 95% CI: 1.06–1.34, *p =* 2.74E-03), acting as mediators. Weighted median and MR-Egger analyses confirmed the IVW, with heterogeneity and pleiotropy detailed in Supplementary Table S5. Scatter plots, leave-one-out plots, and funnel plots for the relevant instrumental variables provided further evidence (Supplementary Figures S26–S33).

We further quantified the mediating effects of these inflammatory proteins (Table 2), finding IL-10 levels mediated the causal relationship between MS, RA, and HT with CRS. Specifically, the mediation effects were −11.65% (95% CI: −26.73 to 3.44) for MS, 10.73% (95% CI: −3.30–24.76) for RA, and 10.48% (95% CI: −1.84–22.79) for HT. For HT and CRS, the mediation proportions for CXCL10 and CD6 were 12.27% (95% CI: −1.95–26.48) and 6.59% (95% CI: −2.99–16.17), respectively. No inflammatory proteins were identified as mediators between ADs and ARS (Supplementary Figure S34).

**TABLE 2:** Mediation analysis of the effect of ADs on CRS risk via potential mediators.

Exposure	Mediator	The Effect of exposure on outcome β(95%CI)	The Effect of exposure on mediator β1 (95%CI)	The Effect of mediator on outcome β2 (95% CI)	Mediated proportion (%) (95%CI)"
Multiple sclerosis	IL-10	0.0357 (0.0069,0.0647)	−0.0301 (−0.0506,-0.0096)	0.1383 (0.0347, 0.2419)	−11.65% (95% CI: −26.73, 3.44)
Rheumatoid arthritis	IL-10	0.0458 (0.0052,0.0864)	0.0355 (0.0141,0.0569)	0.1383 (0.0347,0.2419)	10.73 (-3.30,24.76)
Hypothyroidism or myxoedema	IL-10	0.0679 (0.0191,0.1167)	0.0515 (0.0230,0.0799 0.1383)	0.1383 (0.0347,0.2419)	10,48 (−1.84, 22.79)
	CXCL10	0.0679 (0.0191, 0.1167)	0.0466 (0.0172, 0.0760)	0.1788 (0.0618, 0.2958)	12.27 (−1.9500, 26.4800)
	CD6	0.0679 (0.0191, 0.1167)	0.0304 (0.0001, 0.0607)	0.1472 (0.0329, 0.2616)	6.59 (−2.99, 16.17)

## Discussion

Employing multiple GWAS datasets, this study leverages Mendelian randomization to shed light on the causal links between ADs and RS. Our genetic analyses identified causal associations between five ADs and CRS: MS, CeD, RA, T1D, and HT. The relationship between CeD and ARS is significant. The role of cytokines has been increasingly recognized in the etiology and progression of ADs and RS, though their exact mechanism within the causal framework between ADs and RS is not fully understood. Given the increased risk of developing RS associated with these ADs, we further employed a large-scale genetic dataset comprising 91 cytokines to explore their role in these relationships.

Evidence suggests a notable link between ADs and CRS, indicating that ADs could serve as risk factors for CRS. A cross-sectional analysis highlighted the disparity in CRS prevalence among various ADs, including multiple sclerosis at 1.4%, ankylosing spondylitis at 6.05%, systemic lupus erythematosus at 3.9%, rheumatoid arthritis at 5.0%, psoriasis at 3.8%, and inflammatory bowel disease at 3.5%. Additionally, this study identified the prevalence of CRS with nasal polyps (CRSwNP) at 9.3% and CRS without nasal polyps (CRSsNP) at 91.7%, analyzing the proportions of CRSwNP and CRSsNP within these ADs [31]. A retrospective study in Asia confirmed the significant correlation between CRS and various ADs, particularly in ankylosing spondylitis, SLE, psoriasis, RA, and Sjögren's syndrome [32]. Our research indicates that, beyond MS and RA, T1D, CeD, and HT are also significantly associated with CRS. It is well recognized that immune dysfunction in diabetes mellitus (DM) patients facilitates infections. A retrospective cohort study of 376 CRS patients found that those with DM were prone to Gram-negative bacterial sinus infections [33]. DM patients are at high risk for mucormycosis, susceptible to severe rhino-orbital-cerebral and pulmonary infections. Hence, prompt diagnosis and management of potential life-threatening fungal infections are crucial for DM patients [22, 23, 34]. Although CeD, as a multisystem autoimmune disease, may cause extraintestinal otolaryngological manifestations [24, 25], current research is insufficient to clarify this relationship, indicating the need for further investigation. Limited studies exist on the relationship between CRS and HT. Existing research has shown that patients with HT experience prolonged mucociliary clearance times [26, 27], and animal experiments have demonstrated nasal mucosal hypertrophy, cilia loss, and inflammation following thyroidectomy [28]. These findings align with our results, suggesting the importance of close monitoring for respiratory diseases and sinus infections in patients with HT.

The association between inflammatory proteins and ADs involves intricate regulation of the immune system and a disruption in self-tolerance. Studies have indicated that the expression of tumor necrosis factor-alpha (TNF-α), interferon-gamma (IFN-γ), and several interleukins (such as IL-1, IL-6, IL-12, and IL-17) is elevated in autoimmune diseases, driving inflammation and tissue damage [18]. These cytokines activate immune cells, leading to self-tissue destruction and disease progression [19]. Concurrently, regulatory cytokines, such as IL-10, IL-4, and TGF-β, suppress immune responses by promoting immune tolerance and limiting inflammatory reactions, thereby protecting tissues [18]. Rhinosinusitis, involving inflammation of the nasal sinus mucosa, can be triggered by infections, allergies, or environmental factors, with the disease mechanism centrally involving the modulation of immune system responses by inflammatory proteins [29].

When examining inflammatory proteins as mediators between ADs and RS, we identified IL-10 as a mediator in the relationships between MS, RA, and HT with CRS. As a key anti-inflammatory cytokine, IL-10 effectively suppresses the production of pro-inflammatory cytokines, including TNF-α and IL-1, by various immune cells, such as macrophages and dendritic cells. It also enhances the development of regulatory T cells, regulating immune reactions accordingly [30, 35]. In MS patients, serum IL-10 secretion decreases before MS relapse and increases during remission [36], partially aligning with our findings of reduced IL-10 levels in MS patients. In animal models of autoimmune encephalomyelitis (EAE), IL-10 deficiency resulted in more severe disease progression [37, 38]. Studies have shown increased concentrations of IL-10 in the serum and synovial fluid of RA patients [39, 40]. In animal studies, IL-10 reduced symptoms of RA in rats, curbed neutrophil penetration and activation within synovial tissues, and diminished the production of pro-inflammatory cytokines such as TNF, IL-1, and IL-6 [41, 42]. These findings and our results underscore IL-10’s undisputed regulatory function as an anti-inflammatory cytokine in arthritis. In patients with autoimmune thyroid disease (AITD), IL-10 mRNA expression was found to increase significantly, decreasing as the autoimmune process subsided [43]. IL-10 mitigates inflammation in early thyroiditis by inhibiting Th1 cytokines like TNF-α and IL-1, while in later stages, Th2 cell activation promotes humoral responses [44, 45]. Changes in IL-10 levels among CRS patients remain contentious [46–49]. Moreover, viral and bacterial infections play a crucial role in exacerbating CRS, where IL-10 demonstrates potential therapeutic value in regulating inflammation and ADs by modulating M2 macrophages and mitigating bacterial inflammatory responses triggered by Toll-like receptor signaling [29, 50, 51]. Therefore, these immunoregulatory functions of IL-10 suggest its potential therapeutic role in preventing inflammatory and autoimmune diseases.

The IL-10 cytokine family, which includes IL-19, IL-20, IL-22, IL-24, and IL-26, is integral to regulating anti-inflammatory, pro-inflammatory, and antiviral responses. Although they were not found to act as mediators in our study, they remain key targets for immunomodulatory therapy [52]. Elevated IL-19 expression has been observed in the mononuclear cells within the synovial fluid of RA sufferers [53]. Antibodies targeting IL-19 have prevented arthritis and osteolysis in animal models by diminishing the secretion of pro-inflammatory cytokines, including TNF-α, IL-1β, IL-6, and RANKL [54]. In psoriasis (PsO), elevated levels of IL-19 have been observed, which induce dysplasia and activation of keratinocytes [55, 56], and also prompts CD8^+^ T cells to generate Keratinocyte Growth Factor [57]. IL-20 is linked with increased inflammatory activity in RA joints, and its antagonists may offer therapeutic benefits [58]; its overexpression causes psoriasis-like skin conditions [59], while blocking its pathway reduces psoriasis symptoms [60]. IL-22 is enhanced during active MS, affecting T cell function and participating in the disease process [61], and by regulating keratinocyte differentiation, it influences the mechanisms of PsO [62]. IL-24 aggravates inflammation in RA and PsO, yet it plays a protective role in inflammatory bowel disease by sustaining epithelial and mucosal integrity, thus displaying a bidirectional regulatory capacity in inflammation [63]. In summary, through their complex pro-inflammatory and anti-inflammatory functions, members of the IL-10 family play a crucial regulatory role in ADs.

Our investigation also reveals a causal connection between CXCL10 and the relationship between HT and CRS, highlighting CXCL10’s critical function in immune cell migration to areas of inflammation or damage, thus contributing to the progression of ADs. Elevated CXCL10 expression in conditions such as RA and AITD is closely associated with disease severity and inflammation levels [64, 65]. By interacting with its receptor, CXCR3, CXCL10 triggers Th1 immune responses, promoting infiltration by inflammatory cells and increasing the production of inflammatory mediators, which aggravates tissue damage [64, 66]. Moreover, airway epithelial cells increase the secretion of inflammatory mediators such as IL-1, IL-6, IL-8, and CXCL10 following infections [67, 68], recruiting inflammatory cells to the inflammation site and intensifying the response [69]. Thus, CXCL10, and its receptor CXCR3, are pivotal in enhancing immune responses and inflammation, presenting new avenues for therapeutic intervention in inflammatory diseases and ADs. Additionally, our study identified CD6 as a significant mediator. Primarily found on T and some B cells, CD6 is integral to the immunoglobulin superfamily [70]. It significantly influences T cell activation by interacting with its ligand ALCAM (CD166) [71], bolstering TCR signal transmission and encouraging T cell activation and proliferation [70]. CD6’s role in immune cell adhesion and migration is vital for inflammation and immune surveillance. With CD6 anomalously expressed in multiple ADs, including MS, SLE, and RA [72], its dysregulated activity could exacerbate inflammatory symptoms and tissue damage. Despite limited direct research on CD6 in HT and CRS, its role in autoimmune processes suggests potential underlying connections that merit further exploration.

Beyond the aforementioned mediatory inflammatory proteins, the significance of other inflammatory proteins in ADs should not be overlooked. For instance, IL-17 levels are elevated in patients with MS, and its inhibitors can mitigate brain damage, underscoring the role of IL-17 in promoting inflammation in the nervous system and exacerbating demyelination and axonal damage [73]. Synovitis in RA is characterized by the continuous influx of immune cells and the production of various pro-inflammatory cytokines such as TNF, IL-1, IL-17, and IL-22, which stimulate bone and cartilage inflammation and damage [74]. In T1D, IL-10, TGF-β, and IL-33 aid in reinstating immune tolerance and shielding β-cells from harm, whereas IL-6, IL-17, IL-21, and TNF accelerate the disease’s advancement [75]. Th17 cells, along with their associated inflammatory cytokines IL-17 and IL-23, play a role in the development of autoimmune thyroid (AITD) [44]. IL-15 levels correlate positively with active lesions in patients with celiac disease (CeD), highlighting its importance in CeD [76]. In multiple sclerosis, CC chemokines such as CCL2 and CCL5 promote central nervous system inflammation by regulating immune cell migration [77]. In autoimmune diseases, fibroblast growth factors (FGFs) and their receptors play key roles in inflammatory responses and tissue repair, such as FGF-23, which promotes pro-inflammatory responses of M1 macrophages during infections [78].

Through the application of Mendelian randomization, this research has adeptly navigated confounders and the issue of reverse causation, achieving credible causal effect estimations from observational data [79]. The use of mixed data MR models, incorporating substantial GWAS datasets, has notably increased the efficiency of the analysis compared to smaller-scale, single data models [80]. The study, however, faces significant limitations, notably its sample composed entirely of European individuals, which narrows the generalizability of its findings, and the persistent heterogeneity that could not be fully eradicated, despite thorough attempts with multiple sensitivity analyses, leaving a risk of diversity. Certain Mendelian randomization findings lack empirical backing and thus necessitate careful interpretation, yet they provide a logical foundation for further research. Summarily, the MR analysis elucidated the causal connections between autoimmune diseases and rhinosinusitis, and the mediating influence of inflammatory proteins between ADs and CRS, offering essential biomarkers and potential targets for deciphering disease mechanisms and crafting therapeutic strategies.

## Data Availability

The original contributions presented in the study are included in the article/Supplementary Material, further inquiries can be directed to the corresponding author.

## Footnotes

1
https://www.finngen.fi/en
