# Supplementary material for: The causal relationship between autoimmune diseases and rhinosinusitis, and the mediating role of inflammatory proteins: a Mendelian randomization study
Source: Exp Biol Med (Maywood). 2024 Jul 22;249:10196. doi: 10.3389/ebm.2024.10196 (PMC11299433; doi:10.3389/ebm.2024.10196)
Supplement: Supplementary file 1 [file DataSheet1.DOCX]

Catalogue

[Supplementary Figure 1 1](#_Toc161703426)

[Supplementary Figure 2 2](#_Toc161703427)

[Supplementary Figure 3 3](#_Toc161703428)

[Supplementary Figure 4 4](#_Toc161703429)

[Supplementary Figure 5 5](#_Toc161703430)

[Supplementary Figure 6 6](#_Toc161703431)

[Supplementary Figure 7 7](#_Toc161703432)

[Supplementary Figure 8 8](#_Toc161703433)

[Supplementary Figure 9 9](#_Toc161703434)

[Supplementary Figure 10 10](#_Toc161703435)

[Supplementary Figure 11 11](#_Toc161703436)

[Supplementary Figure 12 12](#_Toc161703437)

[Supplementary Figure 13 13](#_Toc161703438)

[Supplementary Figure 14 14](#_Toc161703439)

[Supplementary Figure 15 15](#_Toc161703440)

[Supplementary Figure 16 16](#_Toc161703441)

[Supplementary Figure 17 17](#_Toc161703442)

[Supplementary Figure 18 18](#_Toc161703443)

[Supplementary Figure 19 19](#_Toc161703444)

[Supplementary Figure 20 20](#_Toc161703445)

[Supplementary Figure 21 21](#_Toc161703446)

[Supplementary Figure 22 22](#_Toc161703447)

[Supplementary Figure 23 23](#_Toc161703448)

[Supplementary Figure 24 24](#_Toc161703449)

[Supplementary Figure 25 25](#_Toc161703450)

[Supplementary Figure 26 26](#_Toc161703451)

[Supplementary Figure 27 27](#_Toc161703452)

[Supplementary Figure 28 28](#_Toc161703453)

[Supplementary Figure 29 29](#_Toc161703454)

[Supplementary Figure 30 30](#_Toc161703455)

[Supplementary Figure 31 31](#_Toc161703456)

[Supplementary Figure 32 32](#_Toc161703457)

[Supplementary Figure 33 33](#_Toc161703458)

[Supplementary Figure 34 34](#_Toc161703459)


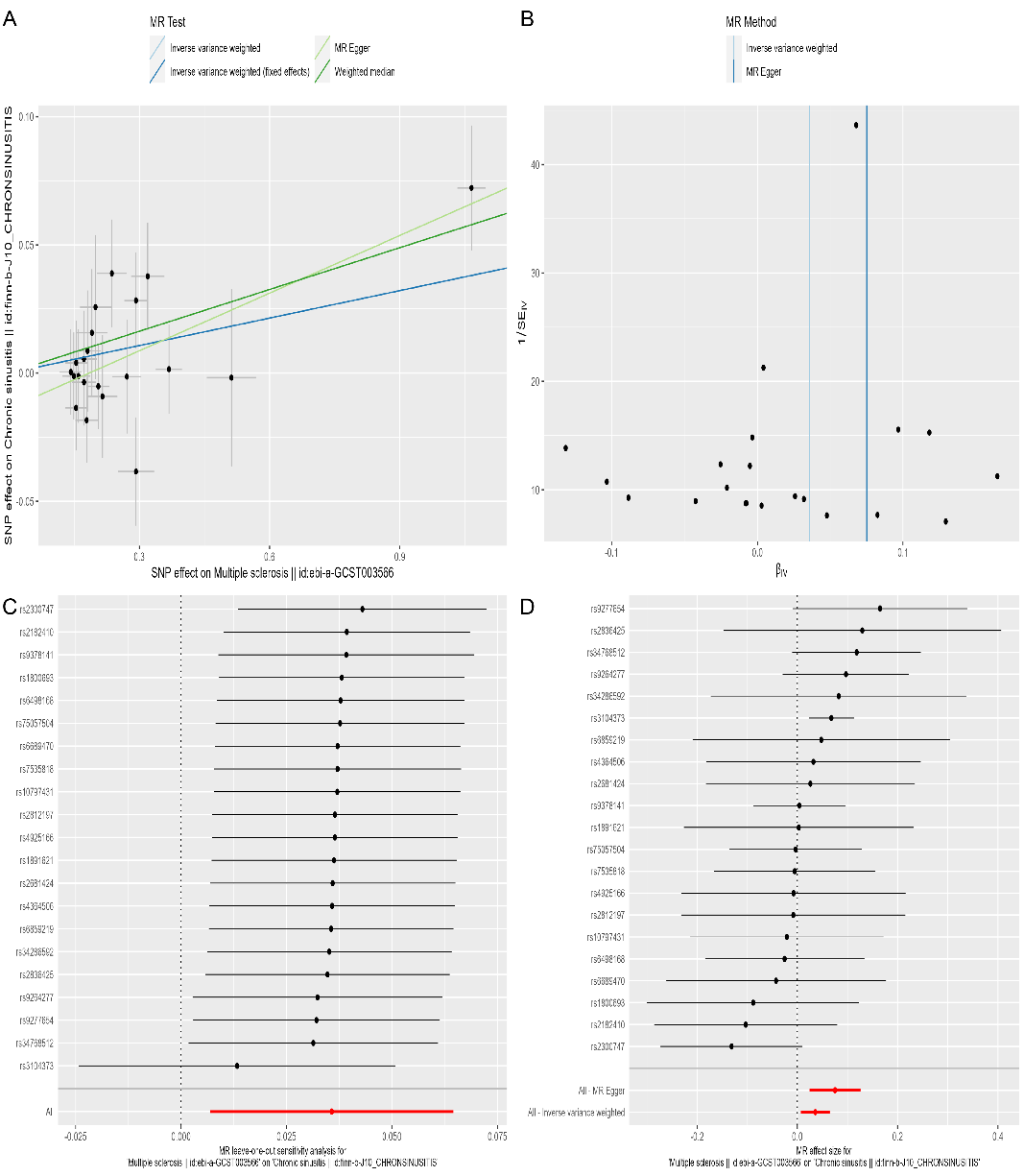
Supplementary Figure 1

MR plots for the causal association of multiple sclerosis on chronic rhinosinusitis. A, Scatter plot: The estimate of the intercept can be interpreted as the average pleiotropy estimate for all single nucleotide polymorphisms (SNPs), while the slope coefficient provides an estimate of the bias in causal effect. B, Funnel plot: the x-axis represents β, and the y-axis represents 1/SE (standard error). C, Leave-one-out sensitivity analysis: Each point and its corresponding line represent the pooled estimate after excluding the respective SNP. D, Forest plot: each dot and its corresponding line represent the effect size and 95%CI. Each dot and its corresponding line represent the pooled estimates after the removal of the corresponding


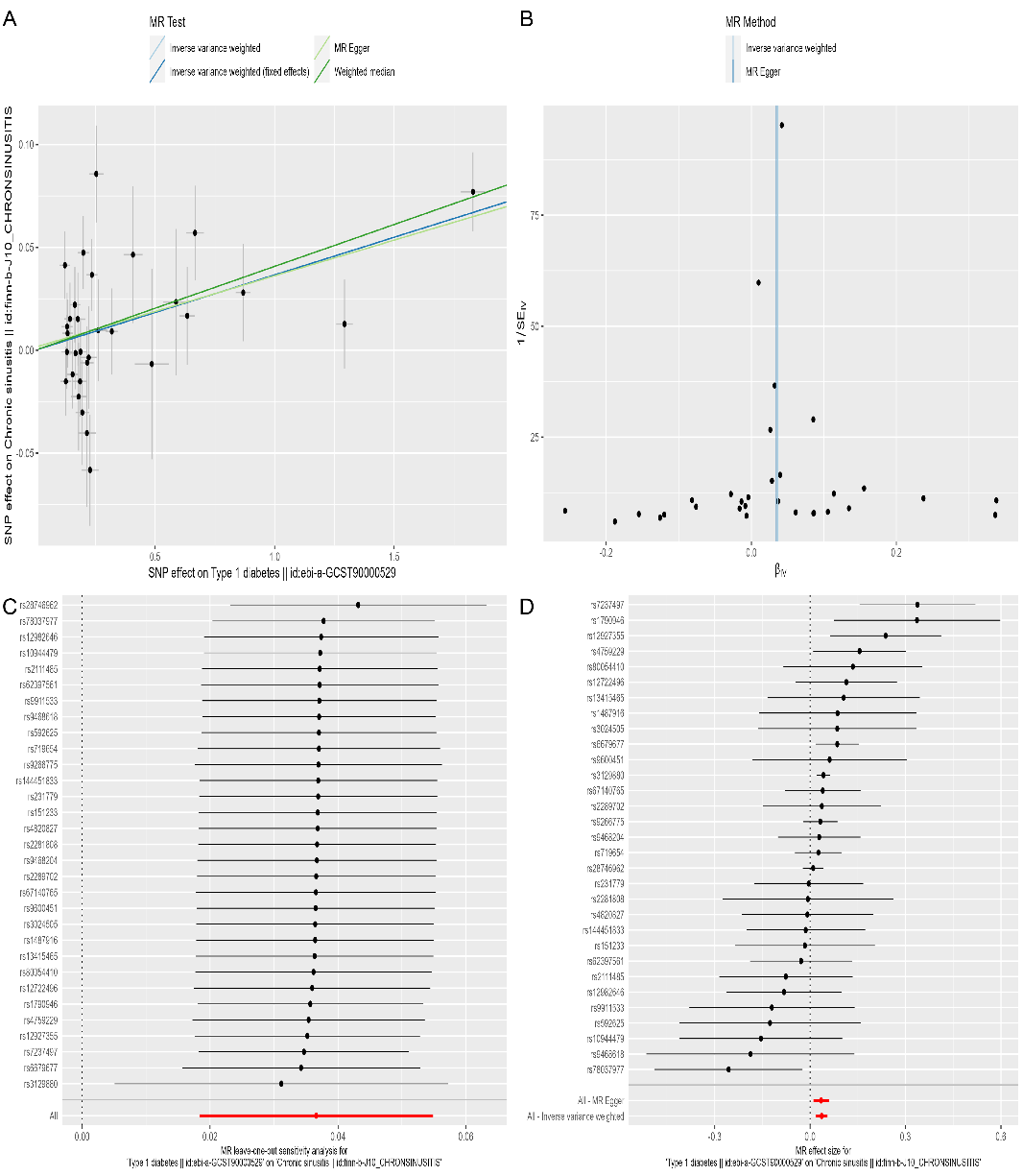
Supplementary Figure 2

MR plots for the causal association of Type 1 diabetes on chronic rhinosinusitis. A, Scatter plots. B, Funnel plot. C, Leave-one-out sensitivity analysis. D, Forest plot.


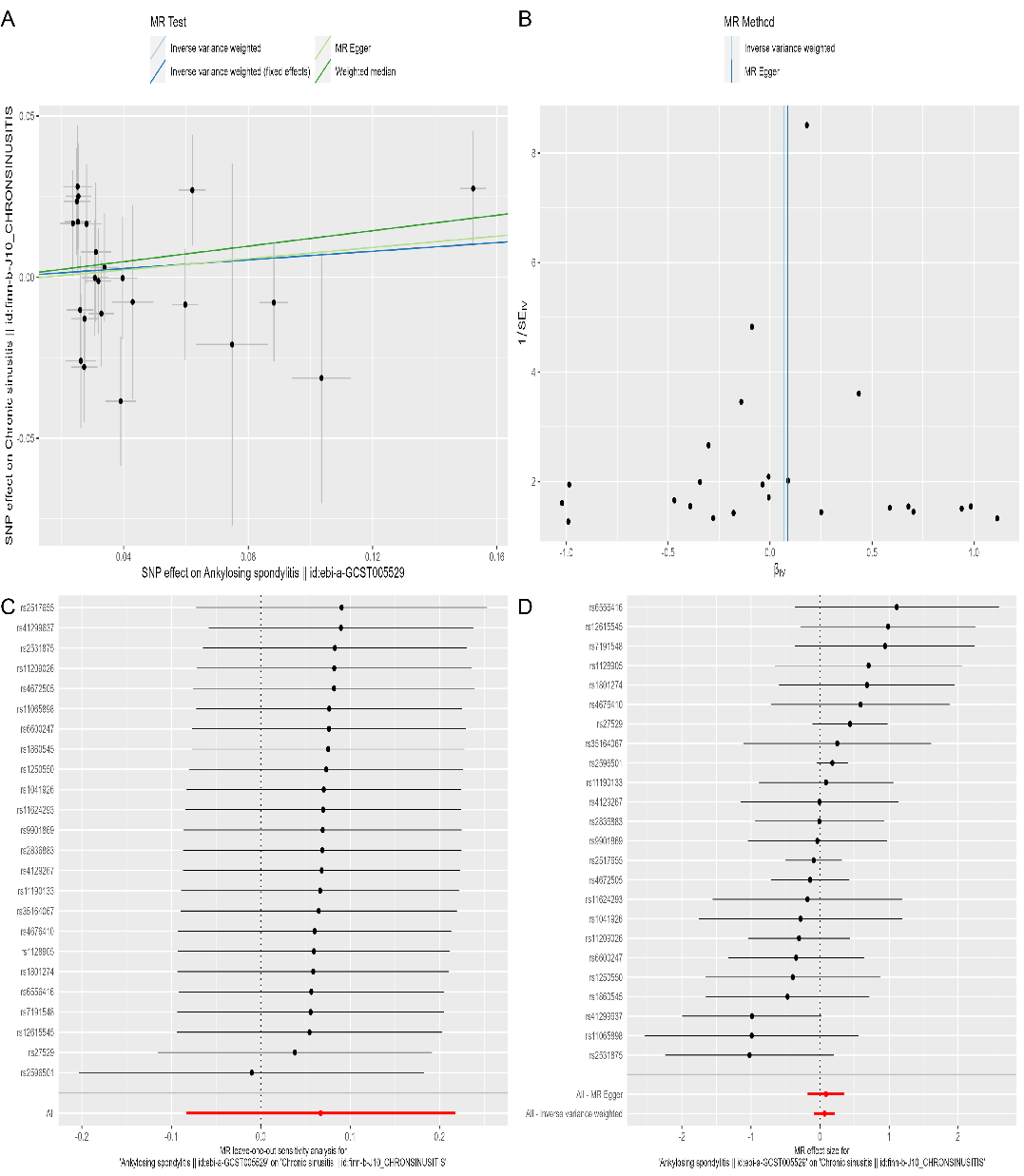
Supplementary Figure 3

MR plots for the causal association of Ankylosing spondylitis on chronic rhinosinusitis. A, Scatter plots. B, Funnel plot. C, Leave-one-out sensitivity analysis. D, Forest plot.


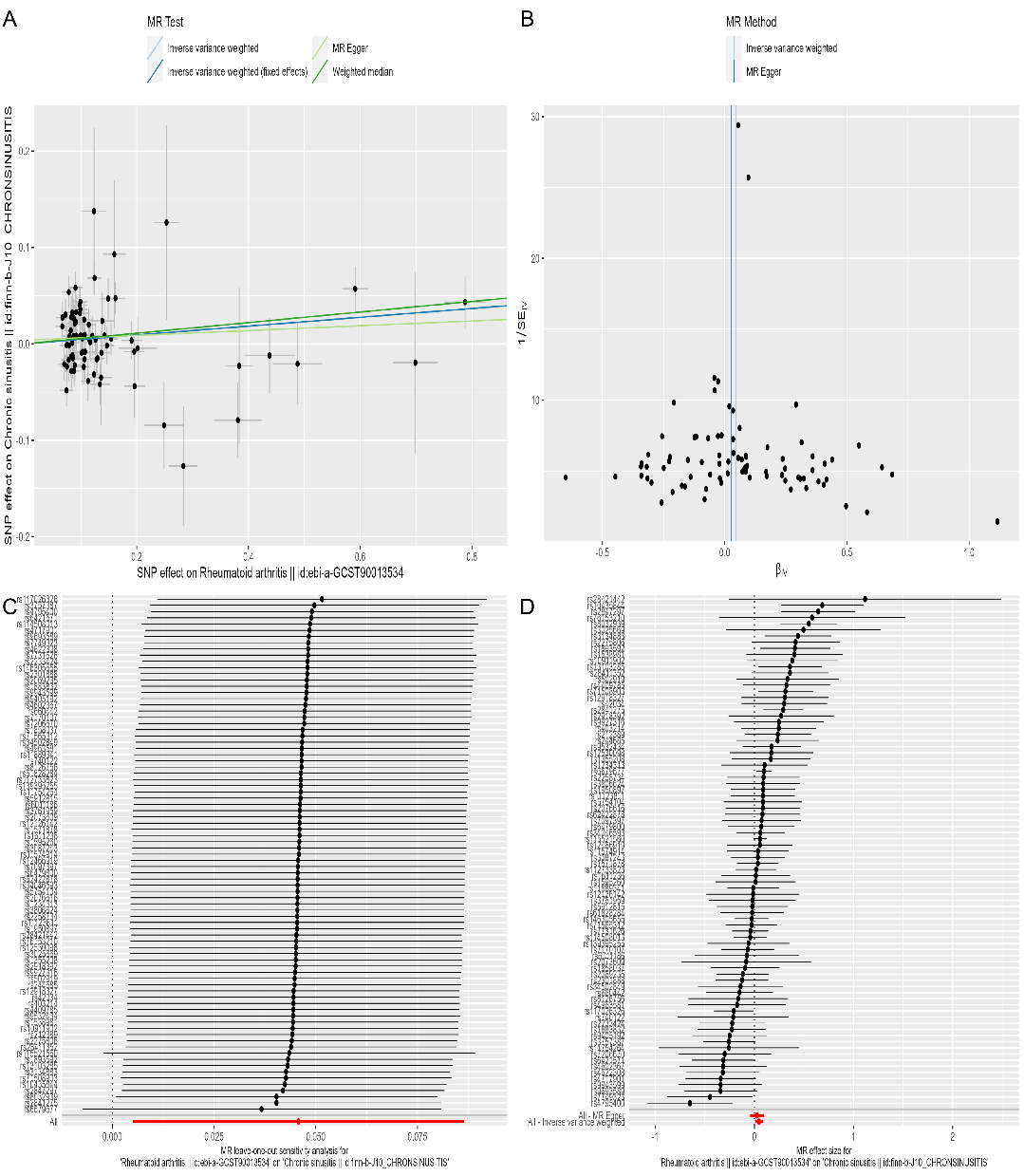
Supplementary Figure 4

MR plots for the causal association of Rheumatoid arthritis on chronic rhinosinusitis. A, Scatter plots. B, Funnel plot. C, Leave-one-out sensitivity analysis. D, Forest plot.


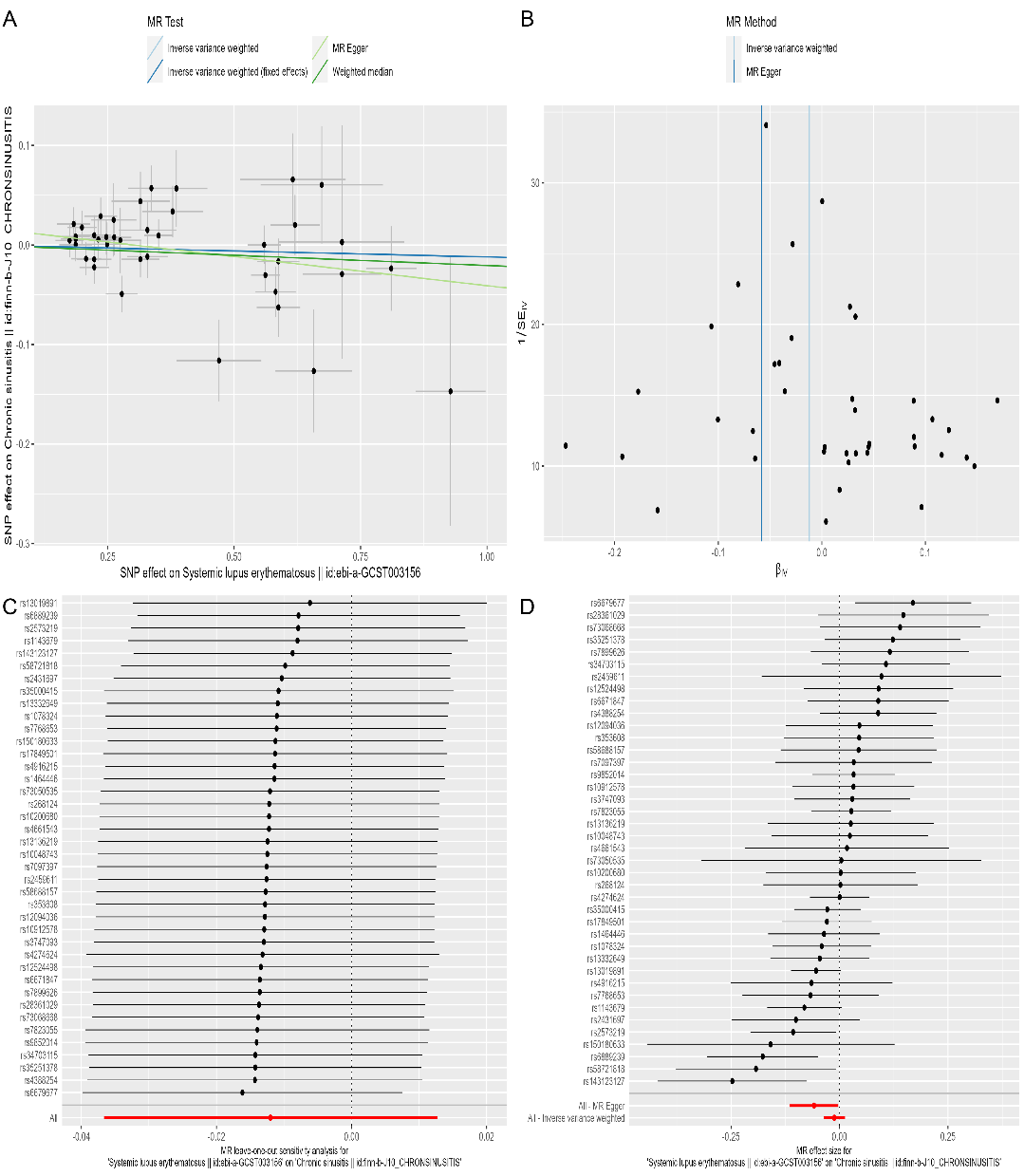
Supplementary Figure 5

MR plots for the causal association of Systemic lupus erythematosus on chronic rhinosinusitis. A, Scatter plots. B, Funnel plot. C, Leave-one-out sensitivity analysis. D, Forest plot.


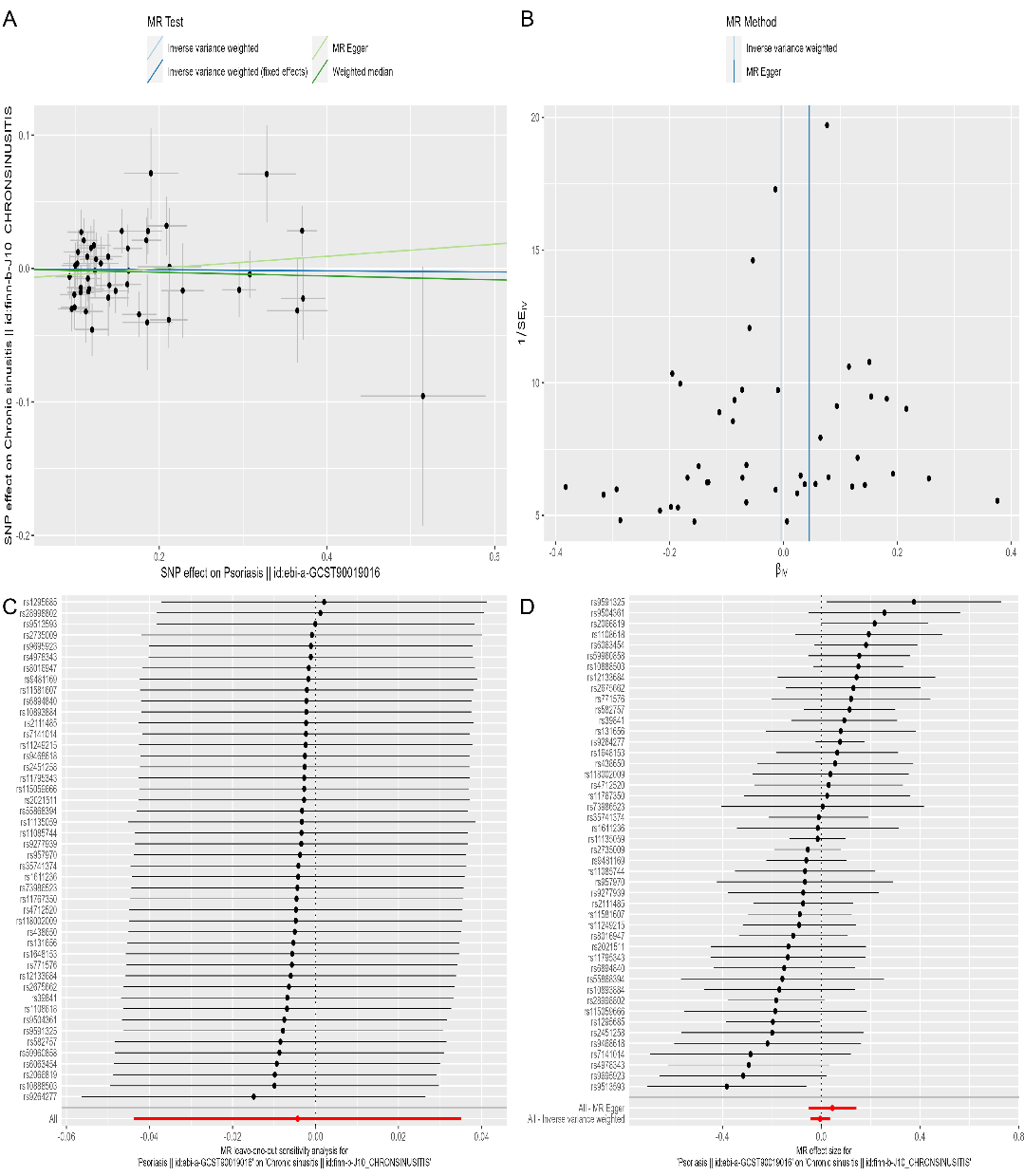
Supplementary Figure 6

MR plots for the causal association of Psoriasison on chronic rhinosinusitis. A, Scatter plots. B, Funnel plot. C, Leave-one-out sensitivity analysis. D, Forest plot.


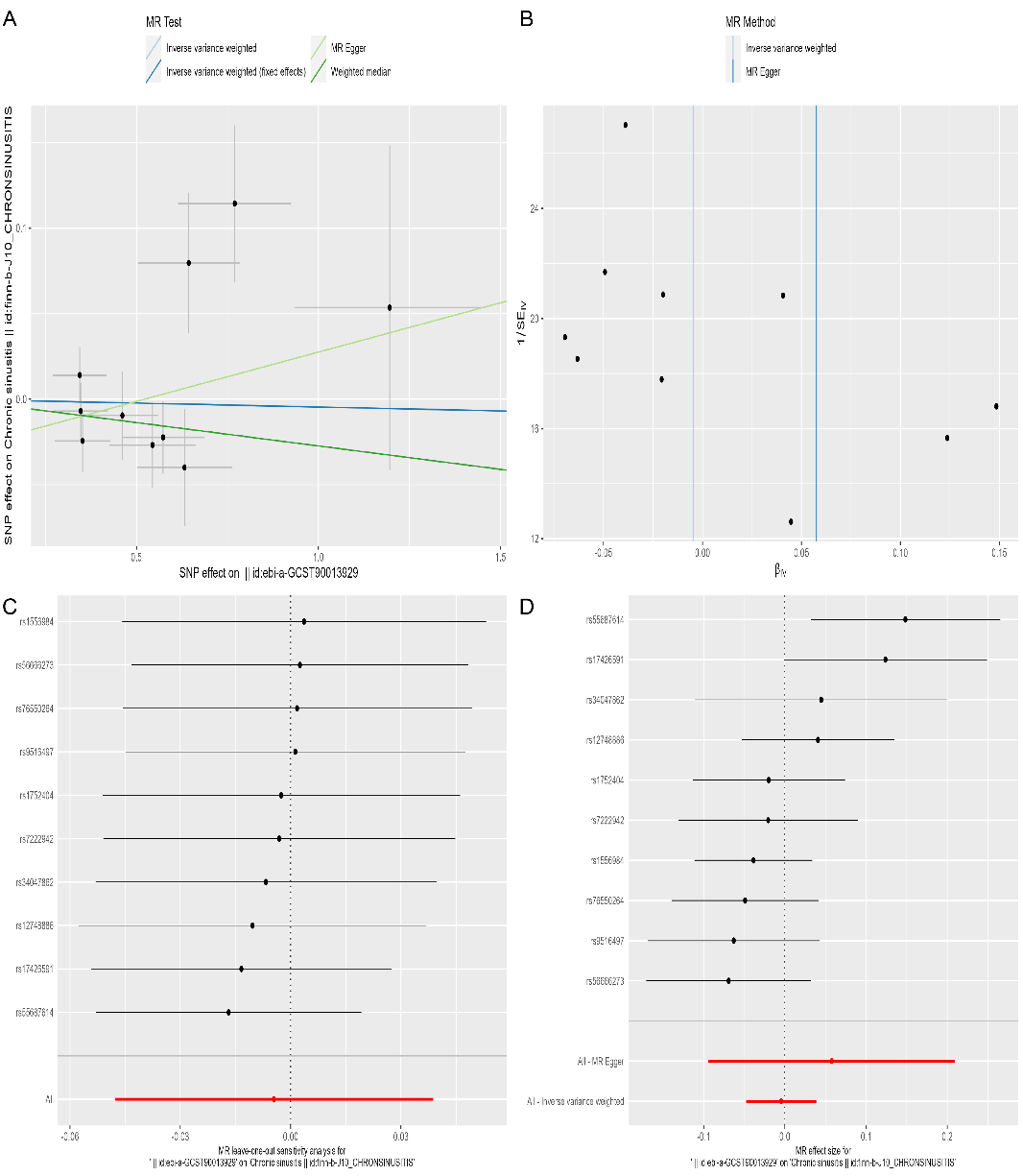
Supplementary Figure 7

MR plots for the causal association of Sjogren's syndrome on chronic rhinosinusitis. A, Scatter plots. B, Funnel plot. C, Leave-one-out sensitivity analysis. D, Forest plot.


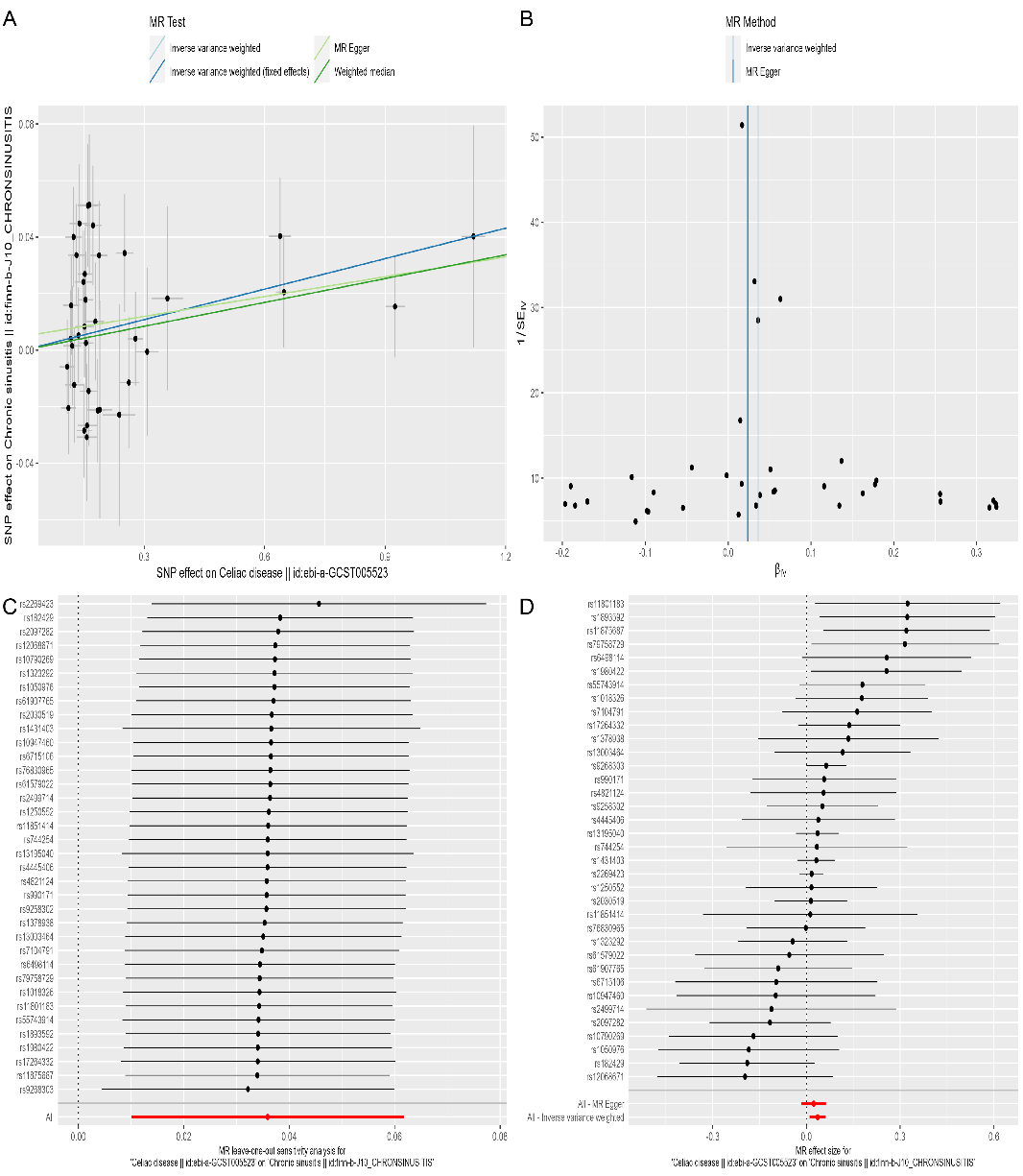
Supplementary Figure 8

MR plots for the causal association of Celiac disease on chronic rhinosinusitis. A, Scatter plots. B, Funnel plot. C, Leave-one-out sensitivity analysis. D, Forest plot.


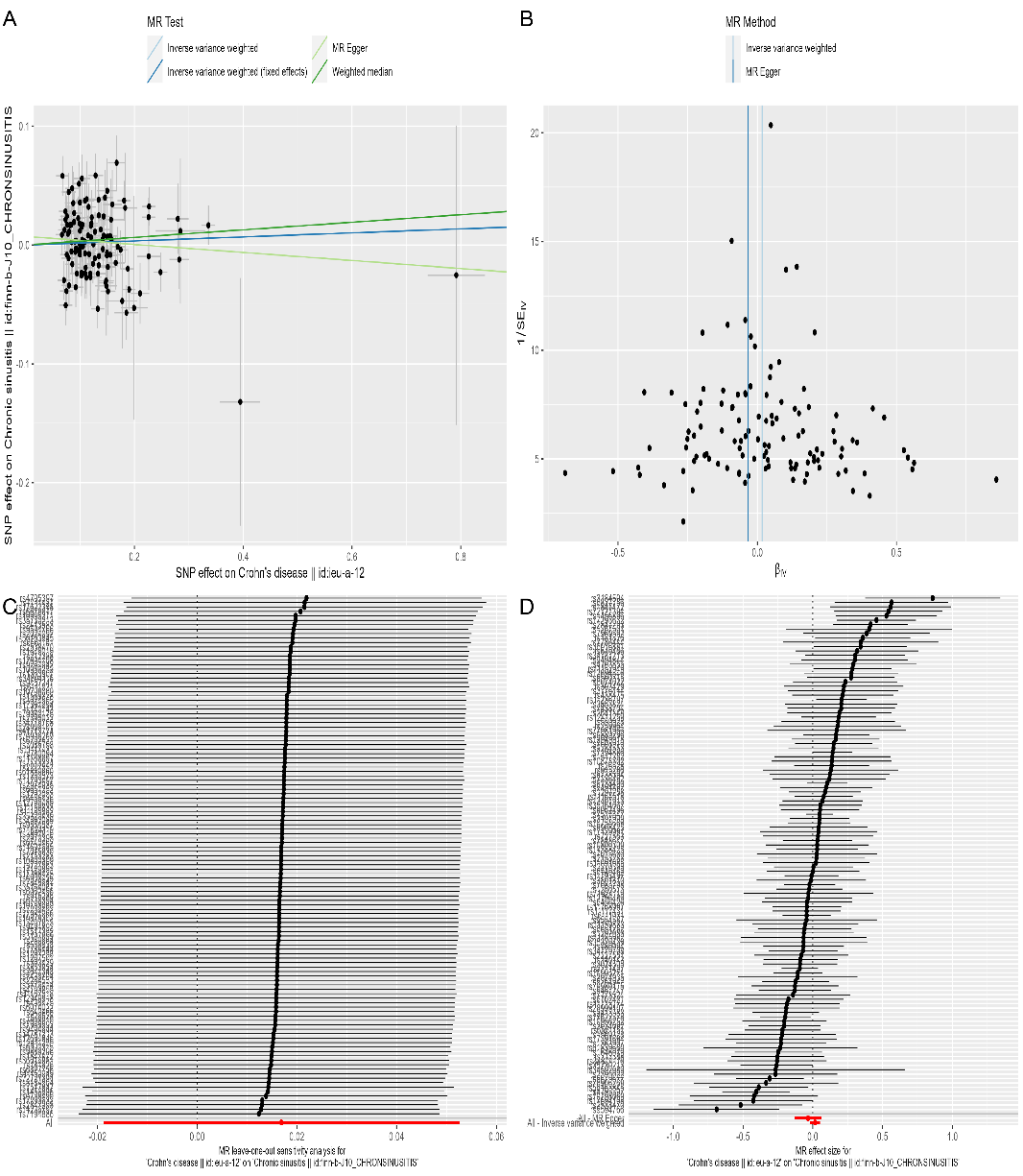
Supplementary Figure 9

MR plots for the causal association of Crohn's disease on chronic rhinosinusitis. A, Scatter plots. B, Funnel plot. C, Leave-one-out sensitivity analysis. D, Forest plot.


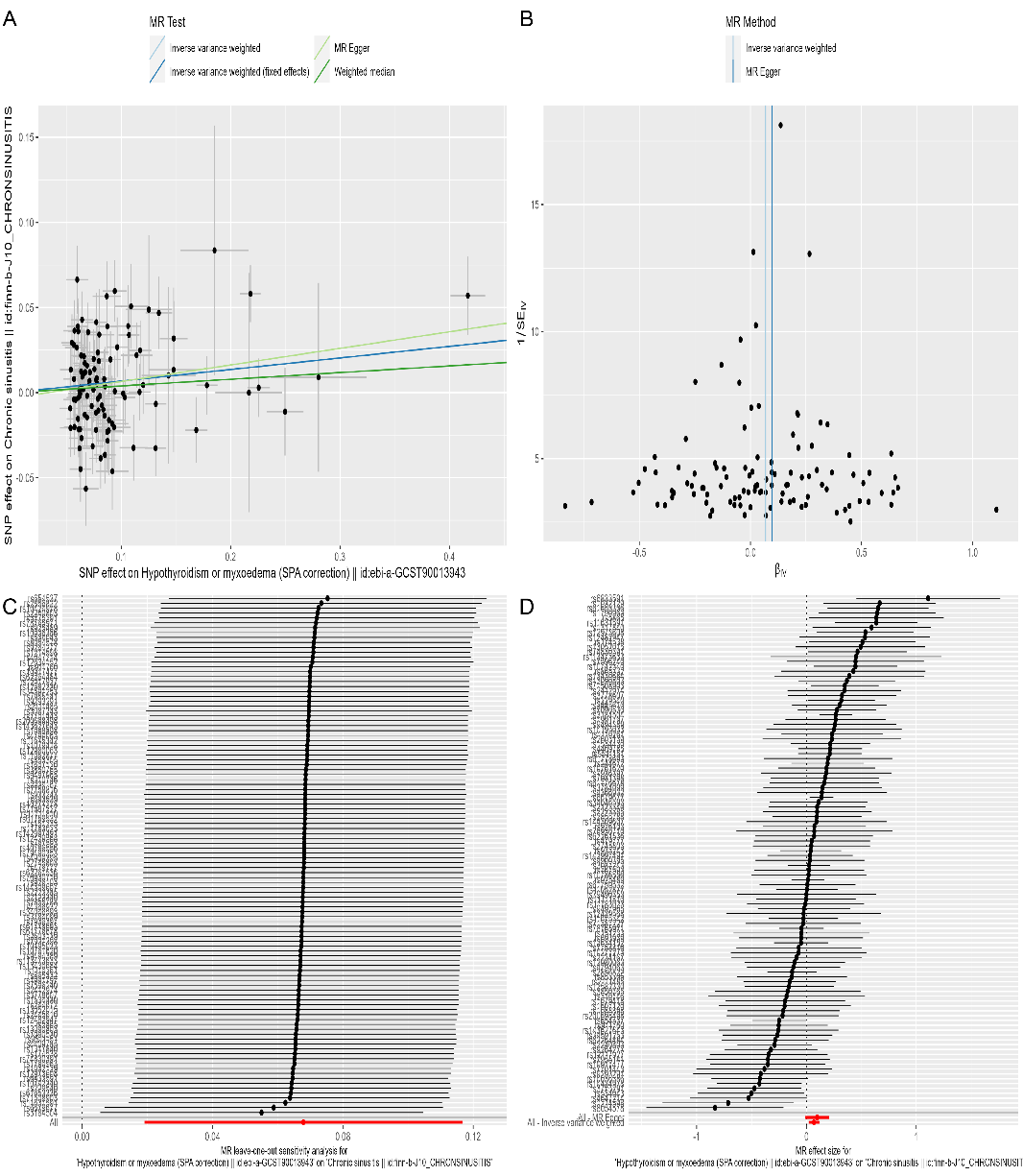
Supplementary Figure 10

MR plots for the causal association of Hypothyroidism or myxoedema on chronic rhinosinusitis. A, Scatter plots. B, Funnel plot. C, Leave-one-out sensitivity analysis. D, Forest plot.


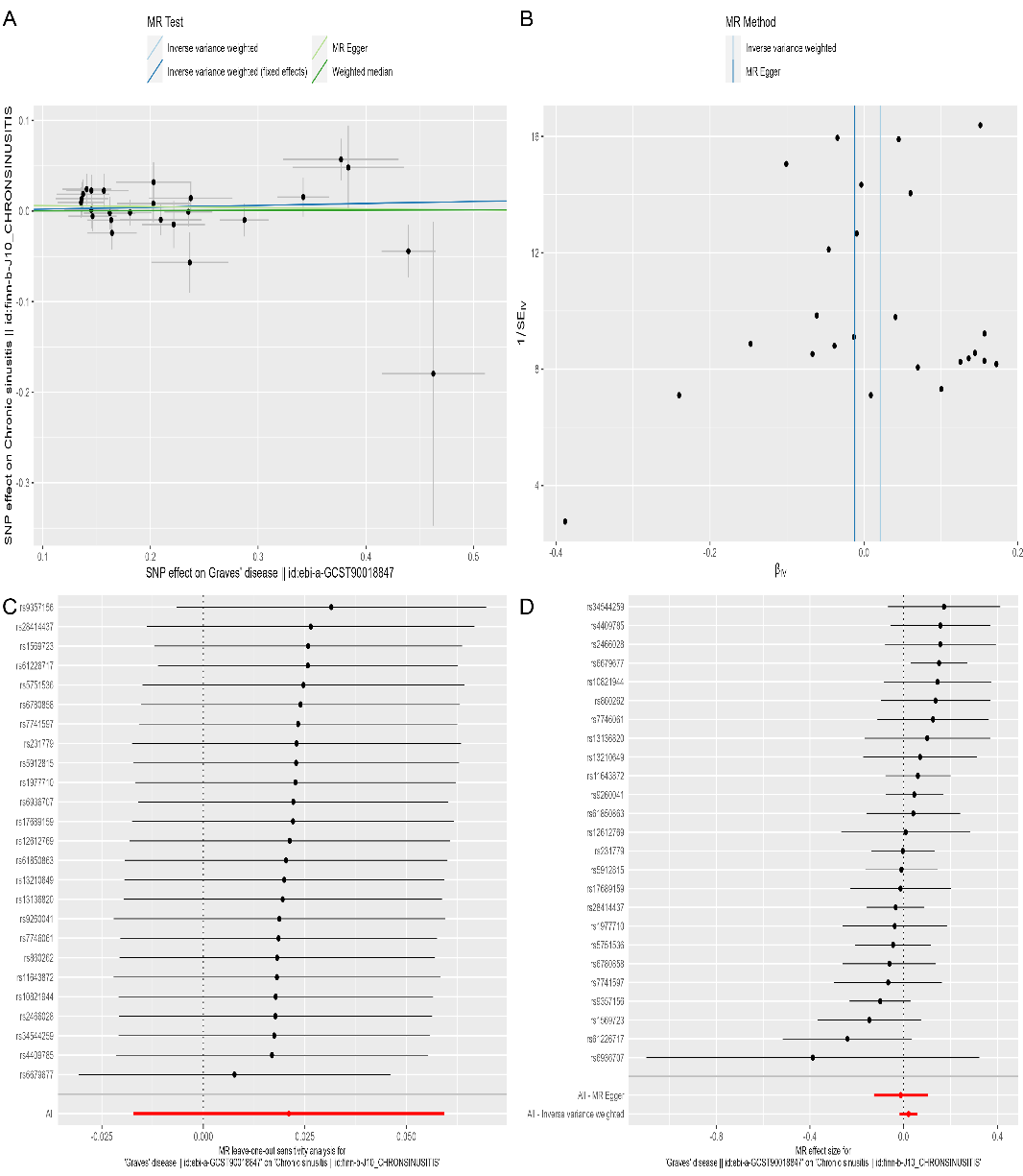
Supplementary Figure 11

MR plots for the causal association of Graves' disease on chronic rhinosinusitis. A, Scatter plots. B, Funnel plot. C, Leave-one-out sensitivity analysis. D, Forest plot.


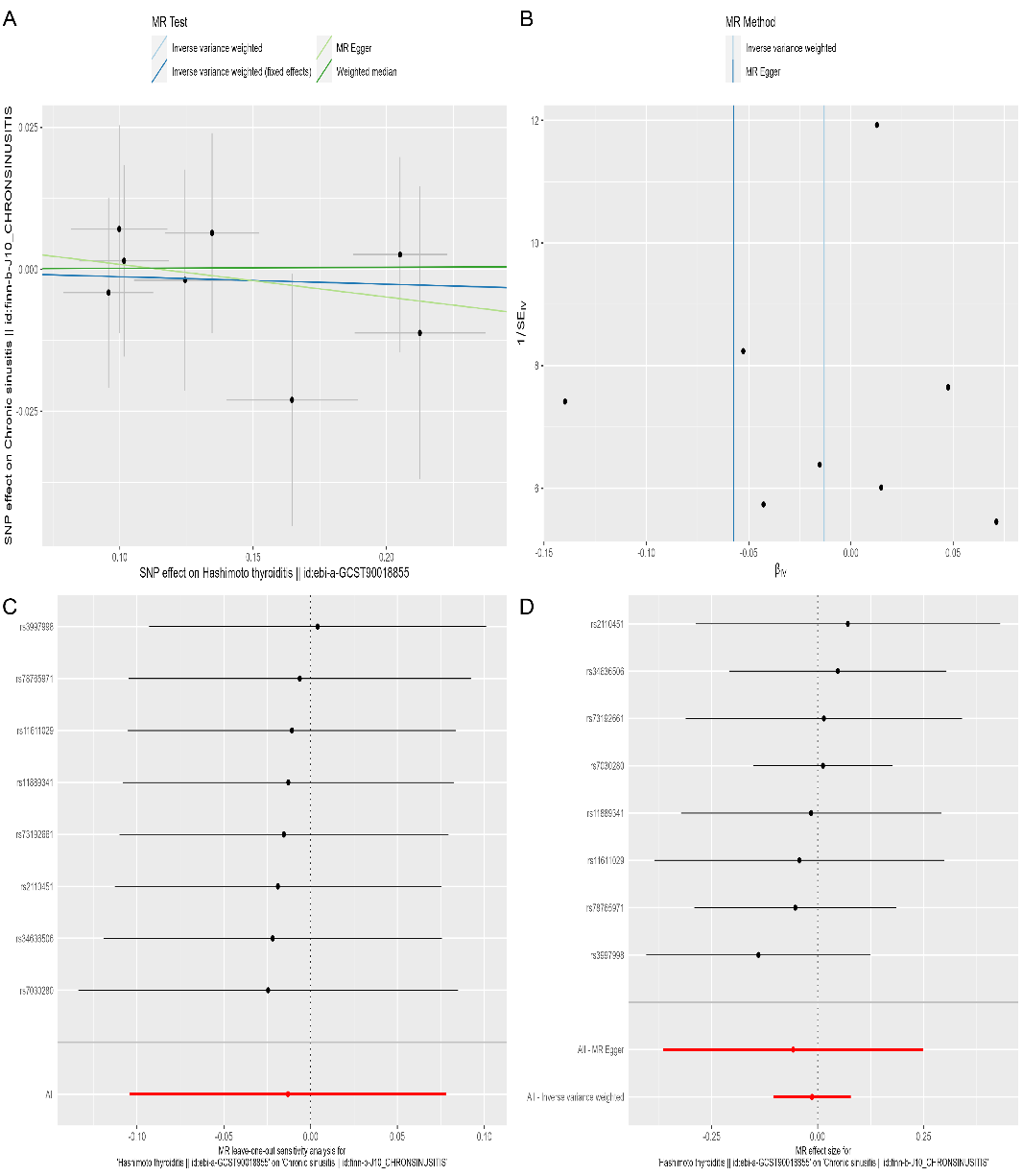
Supplementary Figure 12

MR plots for the causal association of Hashimoto thyroiditis on chronic rhinosinusitis. A, Scatter plots. B, Funnel plot. C, Leave-one-out sensitivity analysis. D, Forest plot.


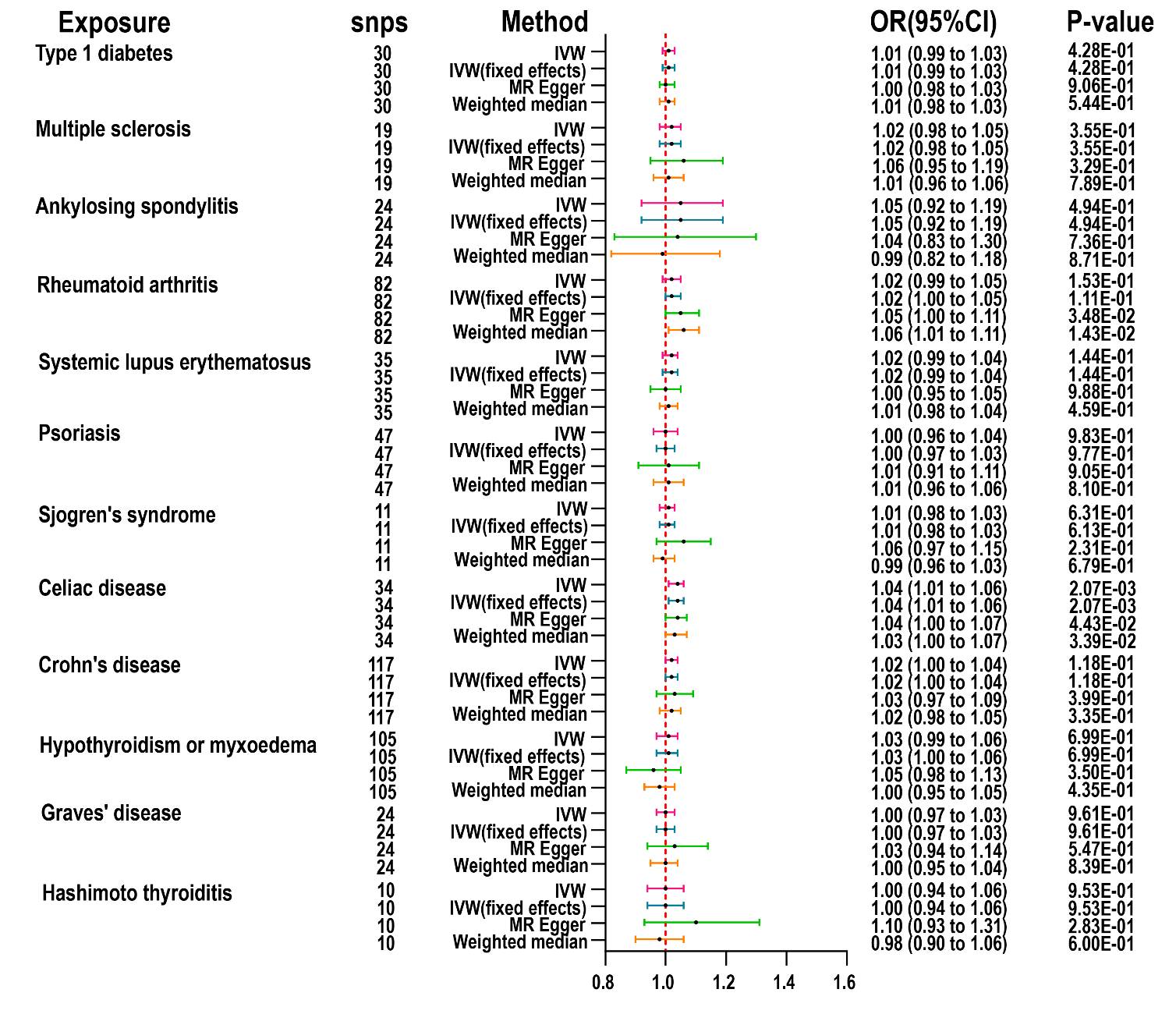
Supplementary Figure 13

Mendelian randomization is used to examine the link between 12 autoimmune diseases and acute sinusitis. IVW, inverse variance weighted; nsnps, number of SNPs ; OR, odds ratio; Cl, confidence interval.


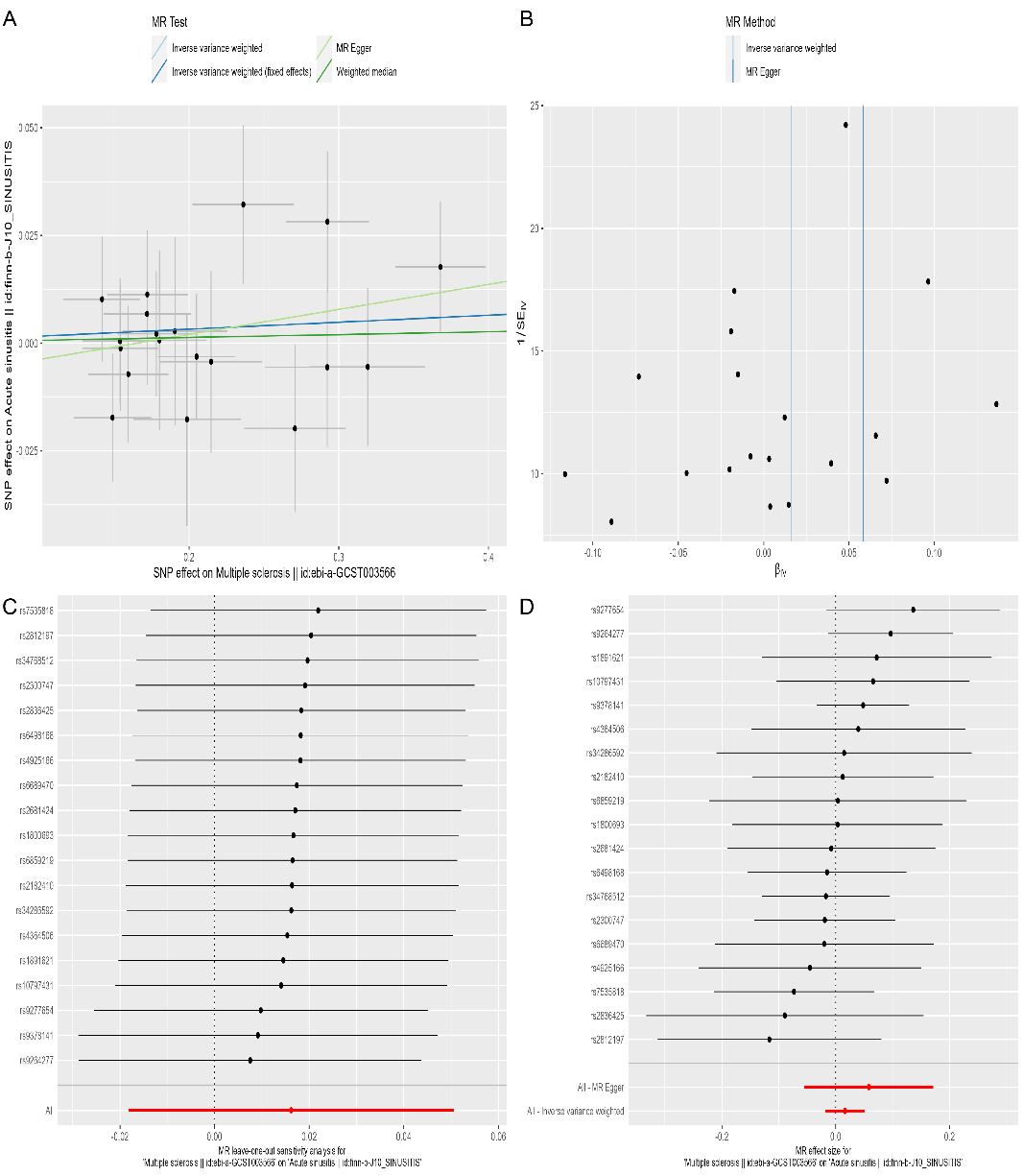
Supplementary Figure 14

MR plots for the causal association of Multiple sclerosis on acute rhinosinusitis. A, Scatter plots. B, Funnel plot. C, Leave-one-out sensitivity analysis. D, Forest plot.


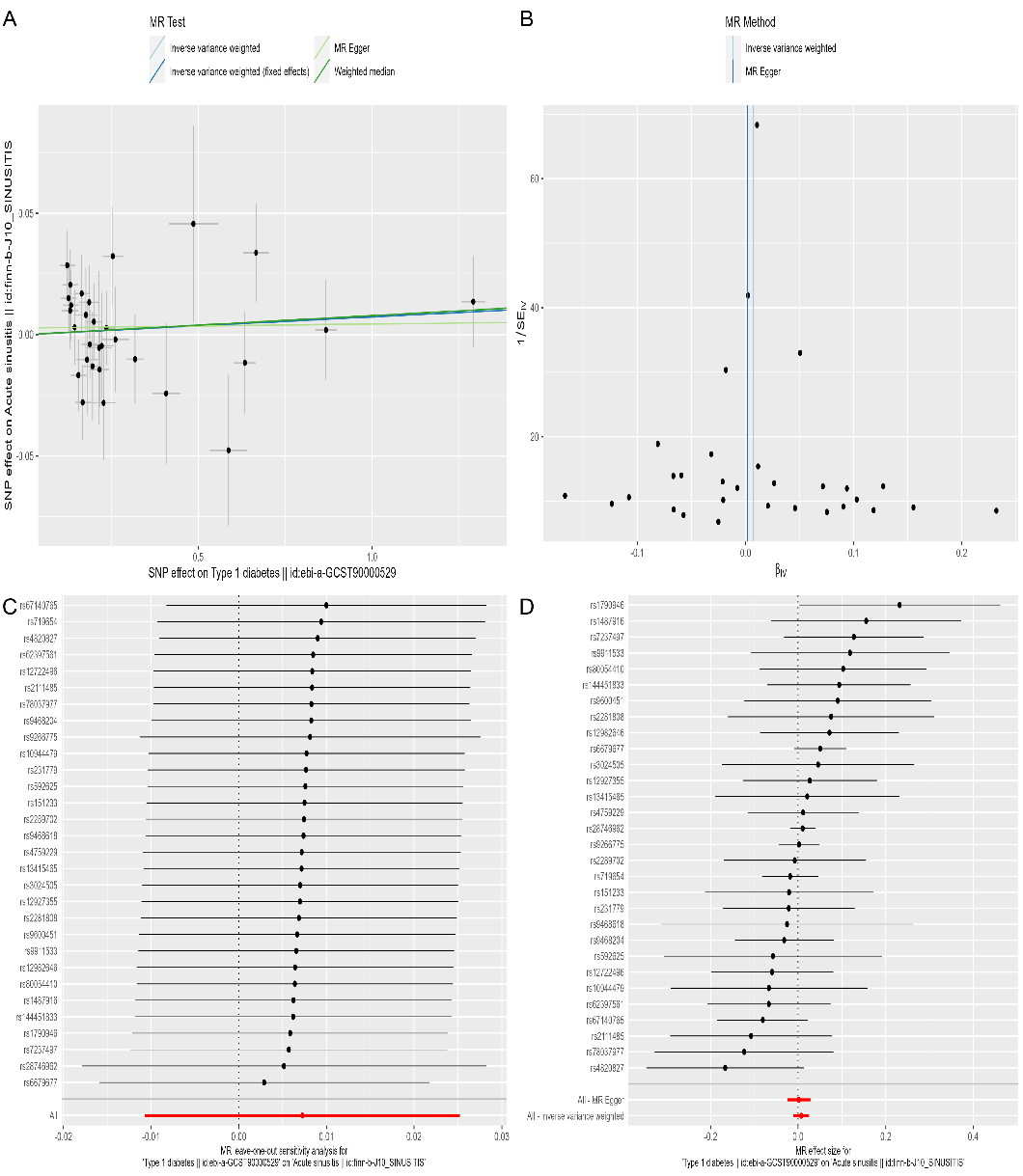
Supplementary Figure 15

MR plots for the causal association of Type 1 diabetes on acute rhinosinusitis. A, Scatter plots. B, Funnel plot. C, Leave-one-out sensitivity analysis. D, Forest plot.


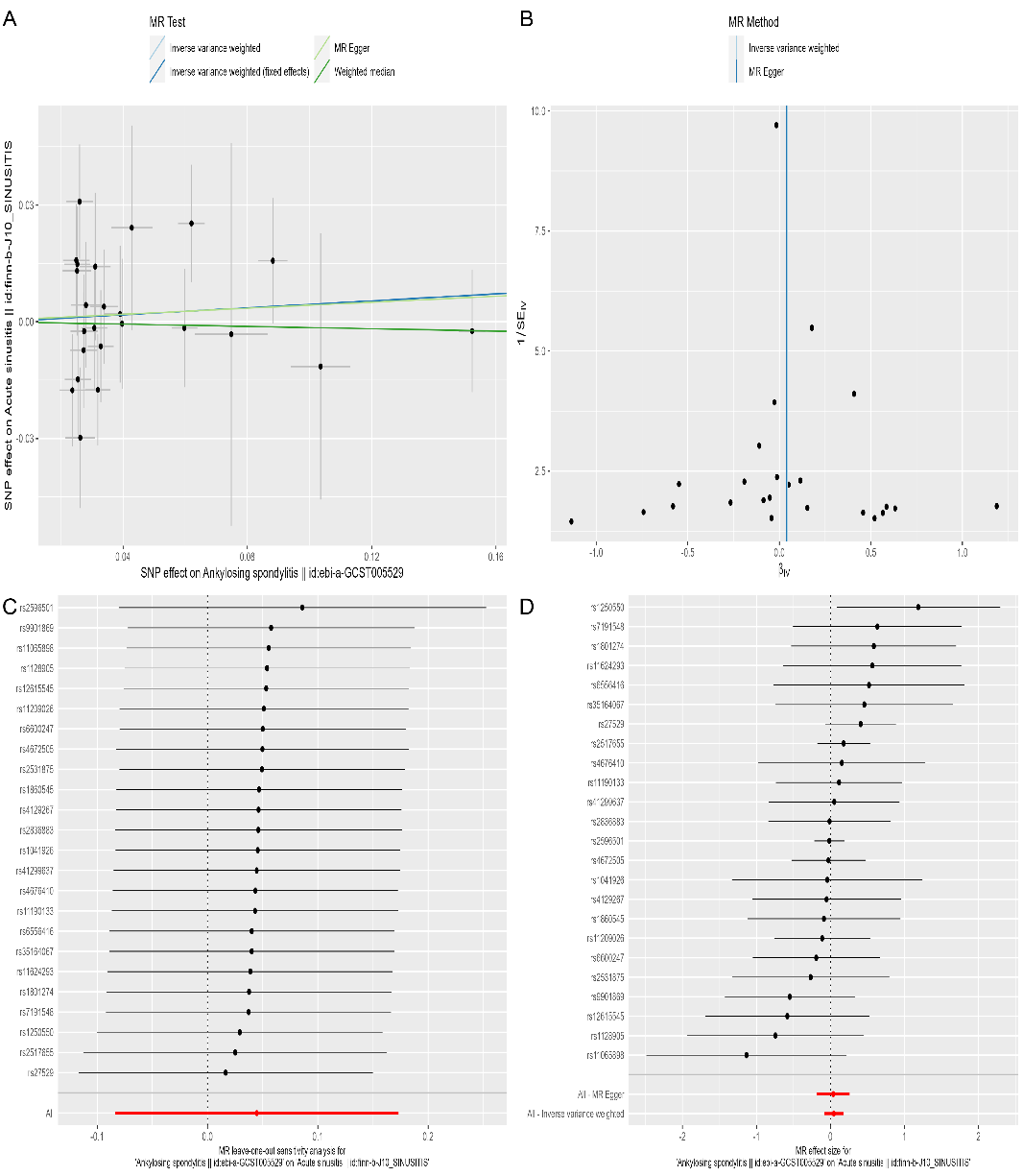
Supplementary Figure 16

MR plots for the causal association of Ankylosing spondylitis on acute rhinosinusitis. A, Scatter plots. B, Funnel plot. C, Leave-one-out sensitivity analysis. D, Forest plot.


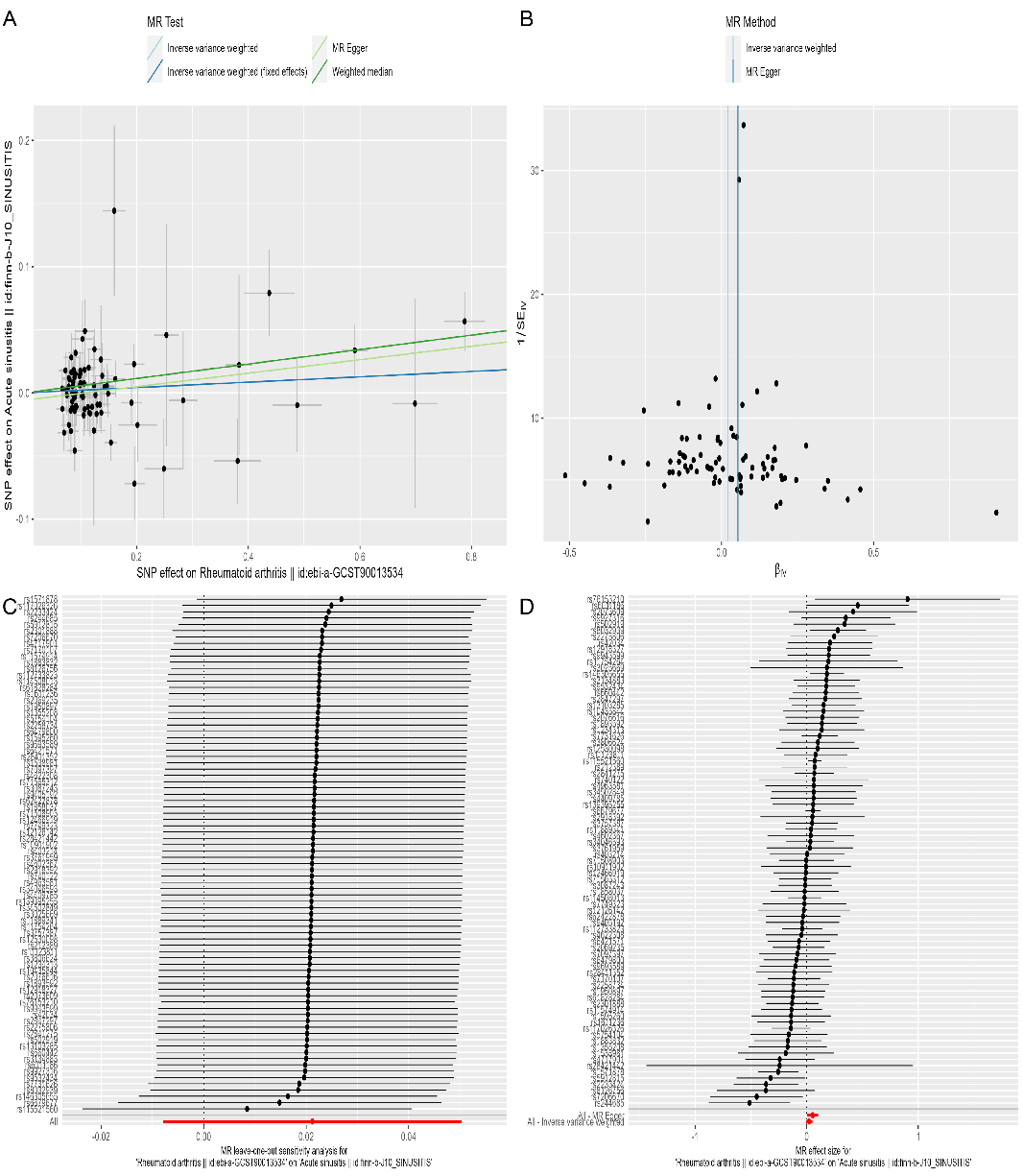
Supplementary Figure 17

MR plots for the causal association of Rheumatoid arthritis on acute rhinosinusitis. A, Scatter plots. B, Funnel plot. C, Leave-one-out sensitivity analysis. D, Forest plot.


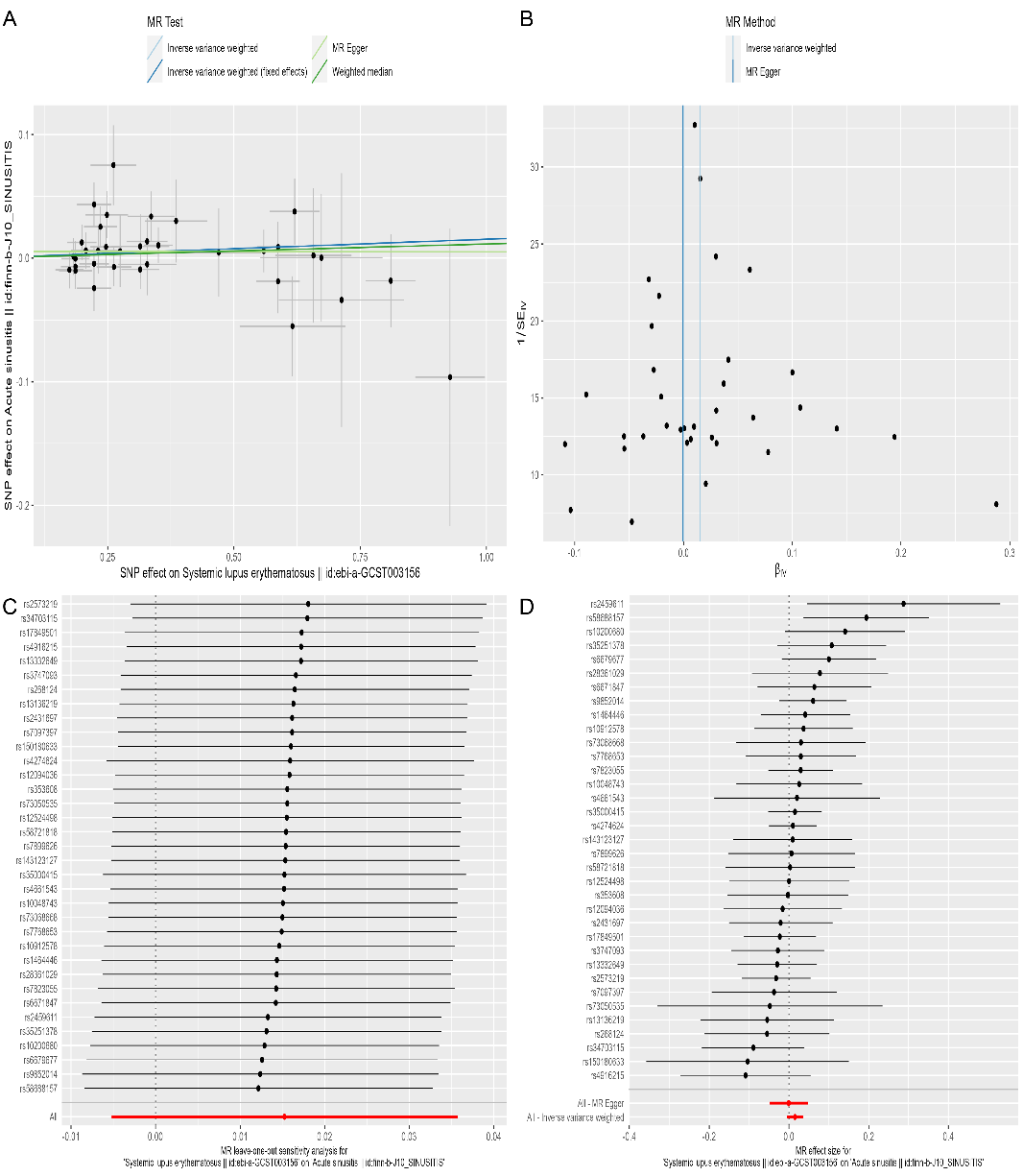
Supplementary Figure 18

MR plots for the causal association of Systemic lupus erythematosus on acute rhinosinusitis. A, Scatter plots. B, Funnel plot. C, Leave-one-out sensitivity analysis. D, Forest plot.


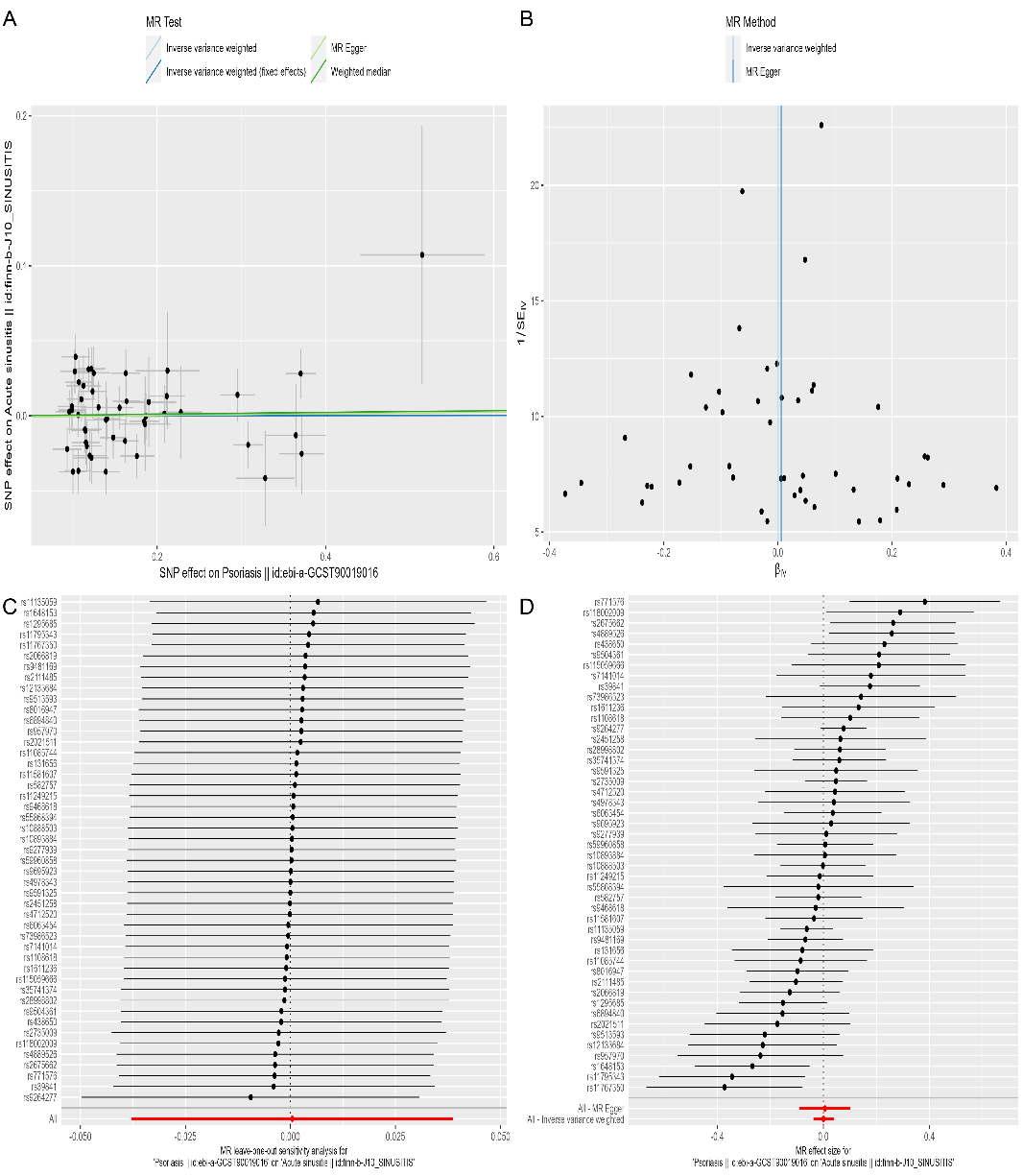
Supplementary Figure 19

MR plots for the causal association of Psoriasis on acute rhinosinusitis. A, Scatter plots. B, Funnel plot. C, Leave-one-out sensitivity analysis. D, Forest plot.


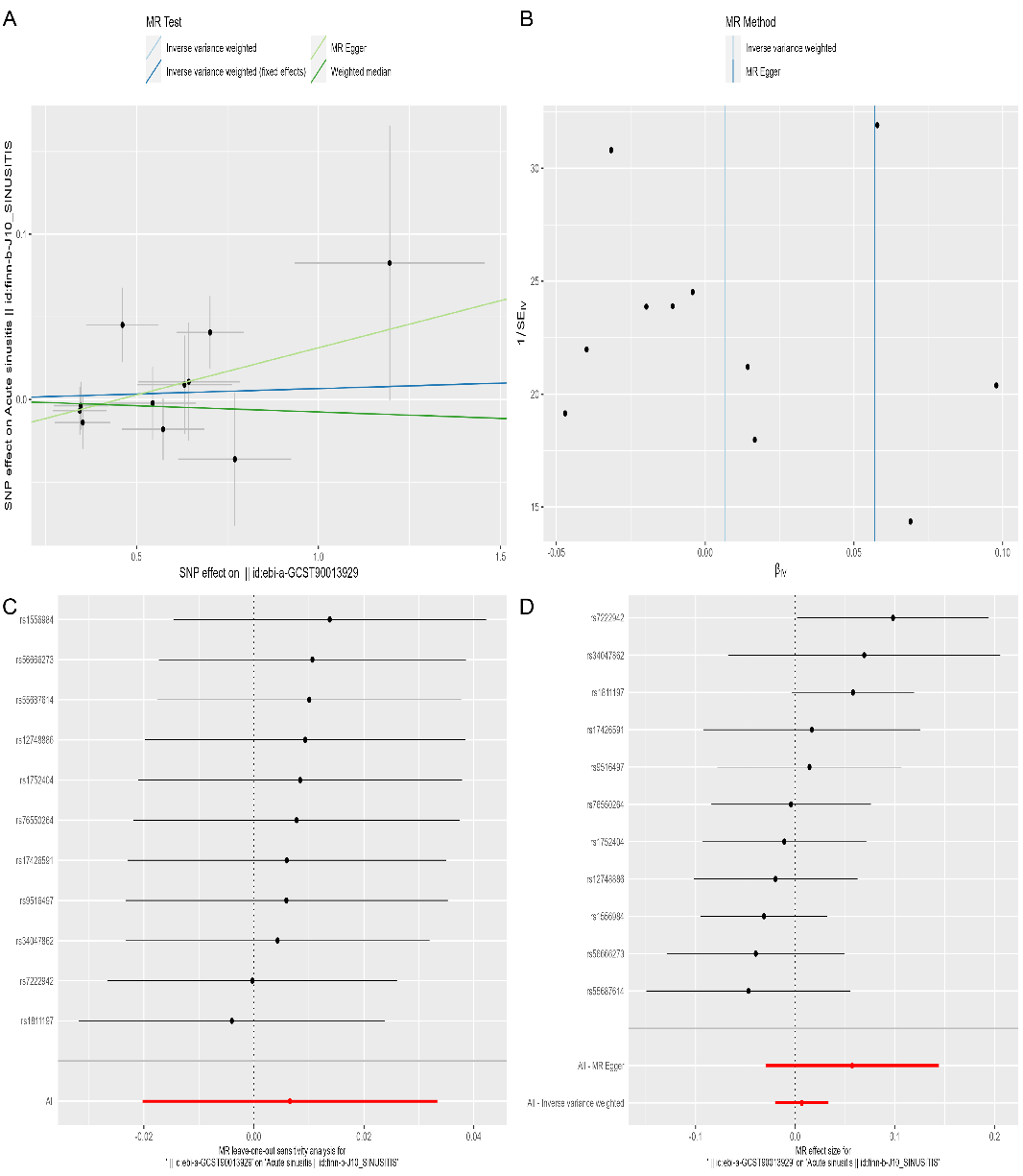
Supplementary Figure 20

MR plots for the causal association of Sjogren's syndrome on acute rhinosinusitis. A, Scatter plots. B, Funnel plot. C, Leave-one-out sensitivity analysis. D, Forest plot.


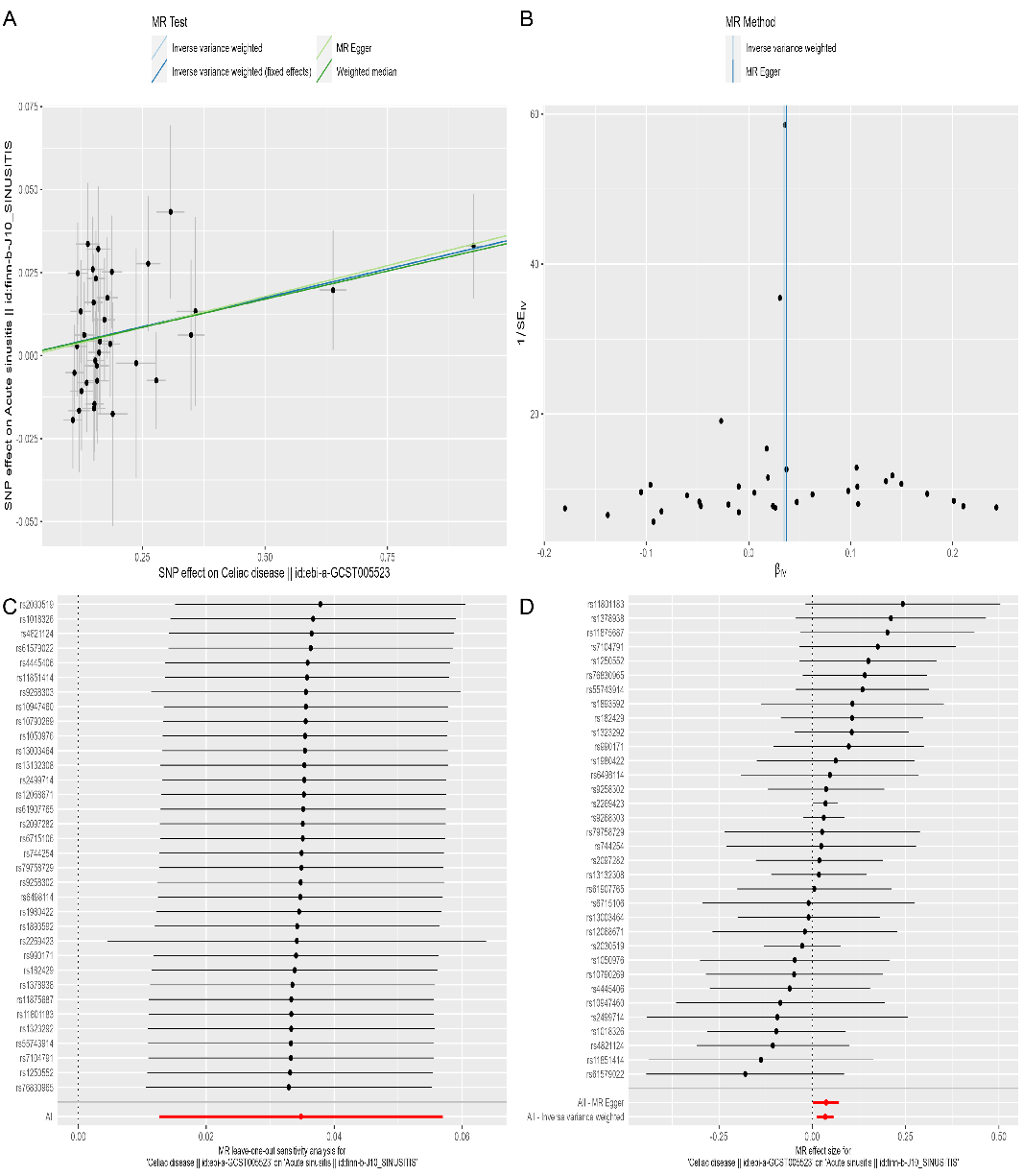
Supplementary Figure 21

MR plots for the causal association of Celiac disease on acute rhinosinusitis. A, Scatter plots. B, Funnel plot. C, Leave-one-out sensitivity analysis. D, Forest plot.


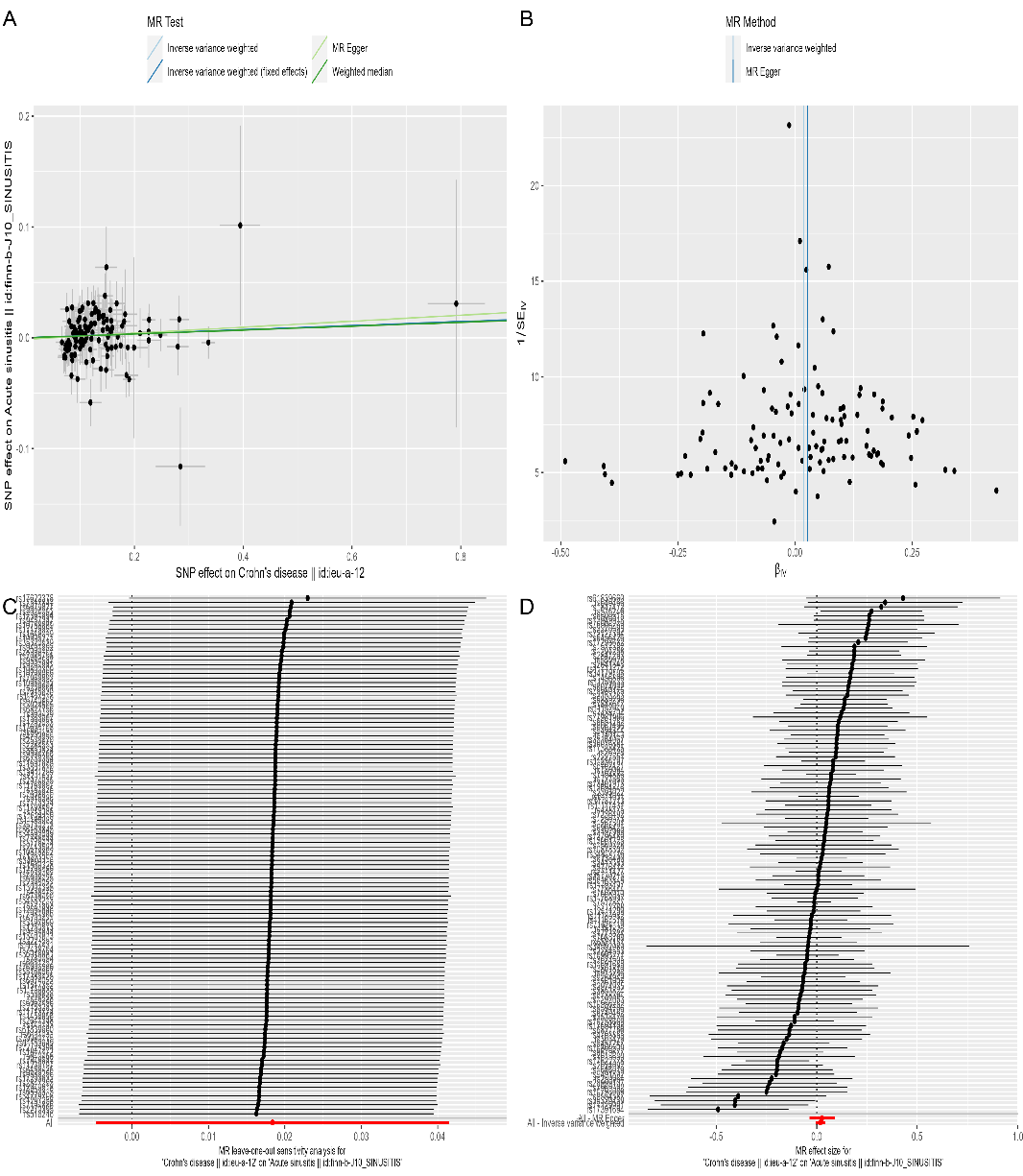
Supplementary Figure 22

MR plots for the causal association of Crohn's disease on acute rhinosinusitis. A, Scatter plots. B, Funnel plot. C, Leave-one-out sensitivity analysis. D, Forest plot.


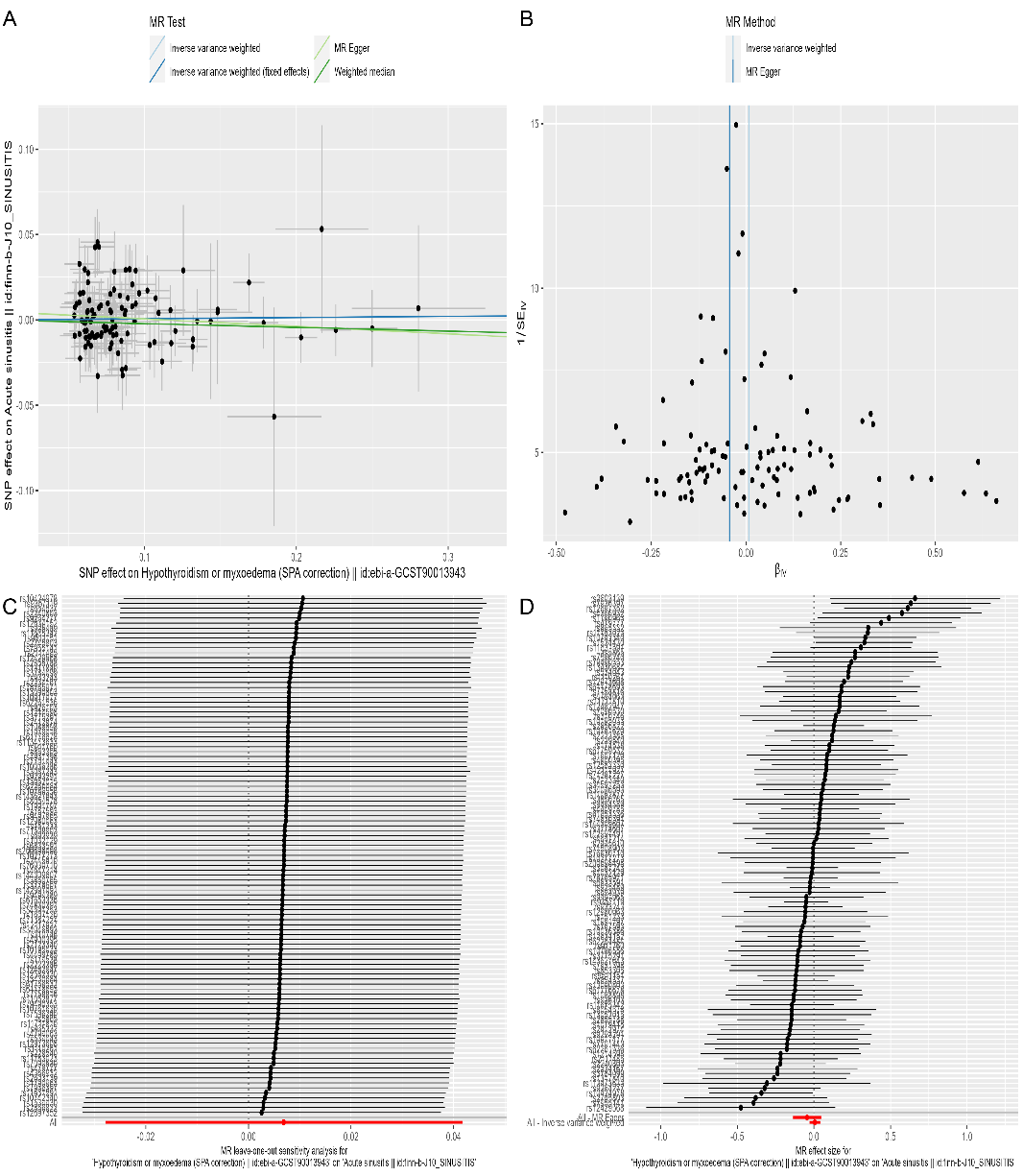
Supplementary Figure 23

MR plots for the causal association of Hypothyroidism or myxoedema on acute rhinosinusitis. A, Scatter plots. B, Funnel plot. C, Leave-one-out sensitivity analysis. D, Forest plot.


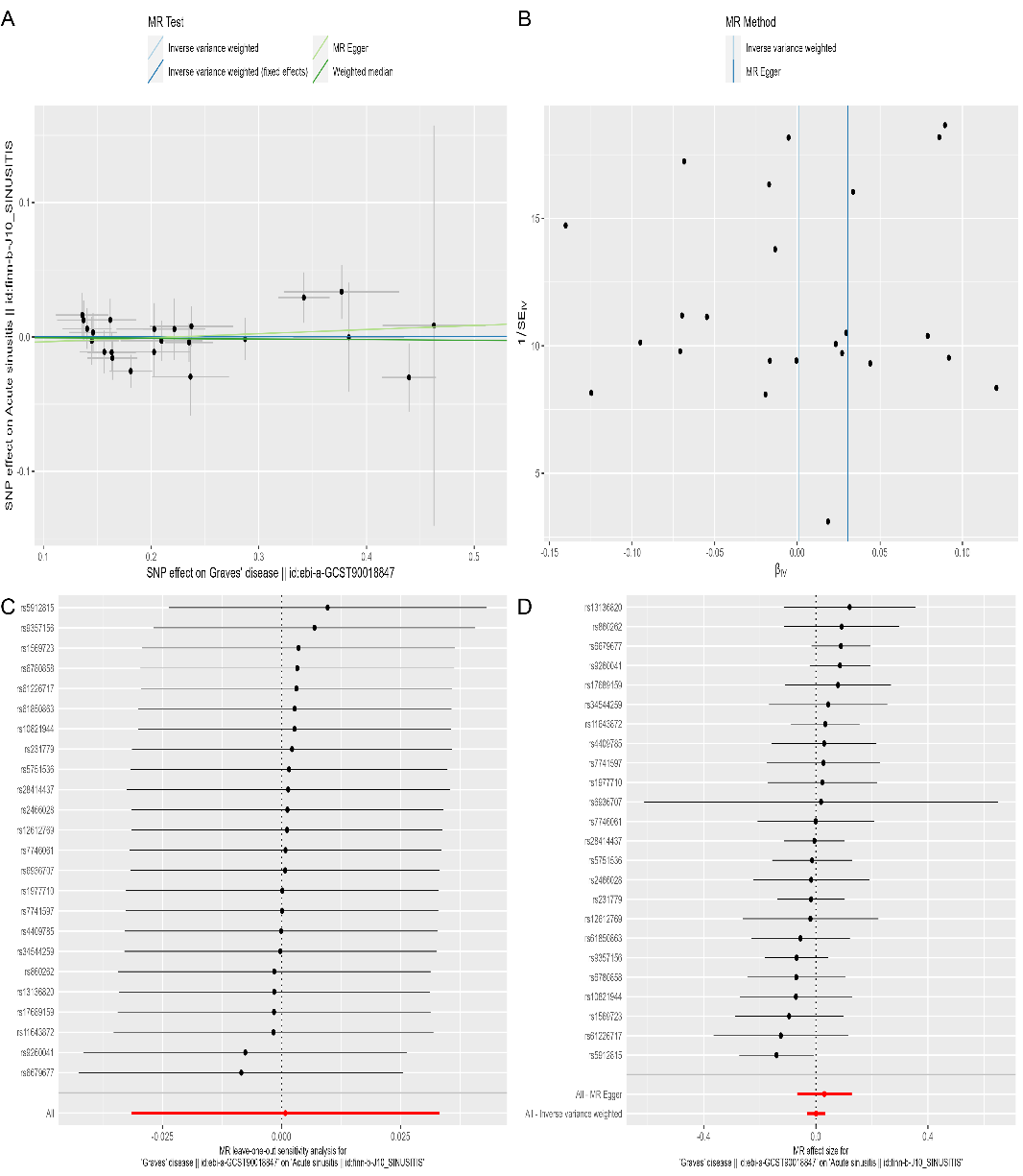
Supplementary Figure 24

MR plots for the causal association of Graves' disease on acute rhinosinusitis. A, Scatter plots. B, Funnel plot. C, Leave-one-out sensitivity analysis. D, Forest plot.


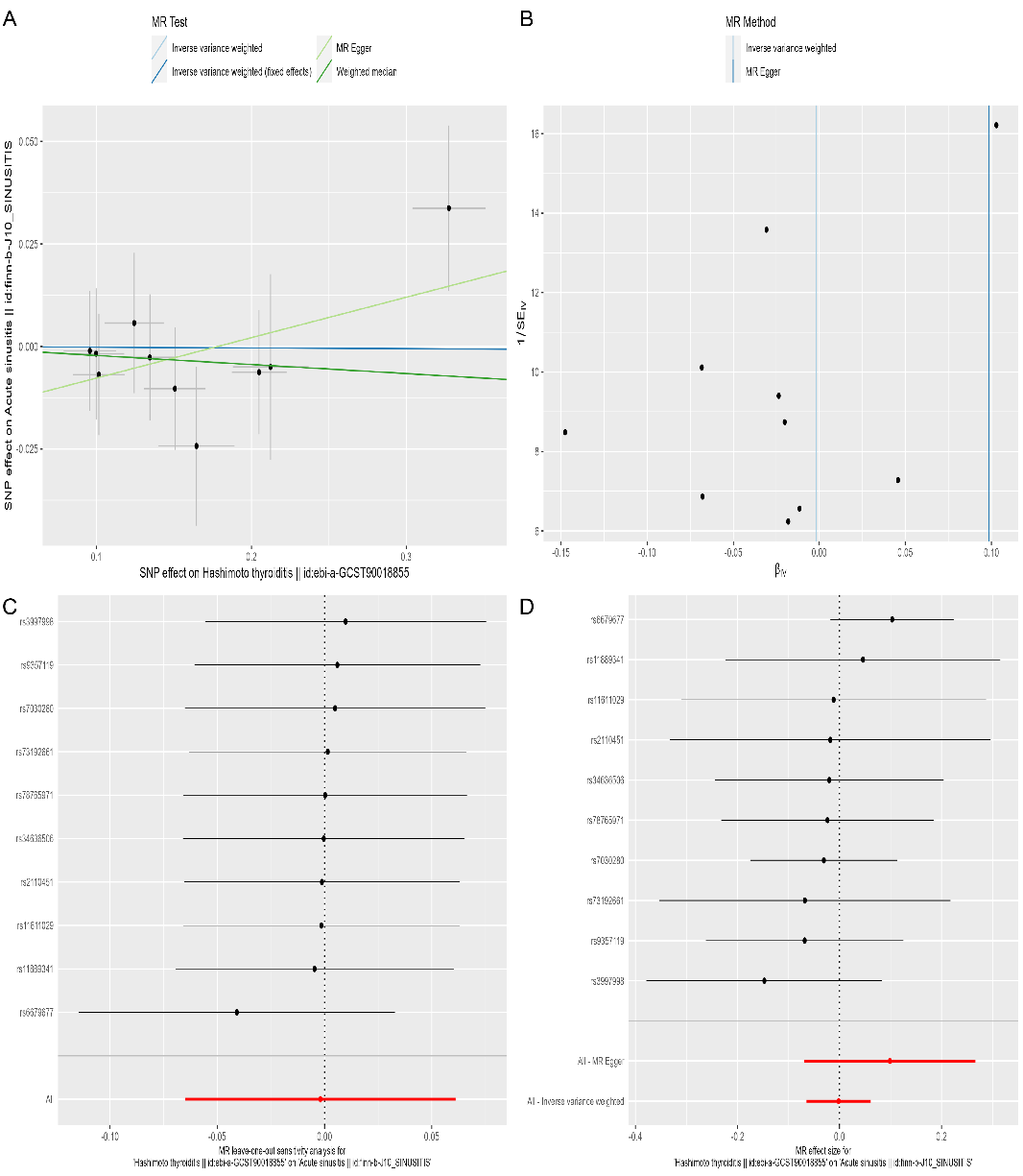
Supplementary Figure 25

MR plots for the causal association of Hashimoto thyroiditis on acute rhinosinusitis. A, Scatter plots. B, Funnel plot. C, Leave-one-out sensitivity analysis. D, Forest plot.

Supplementary Figure 26


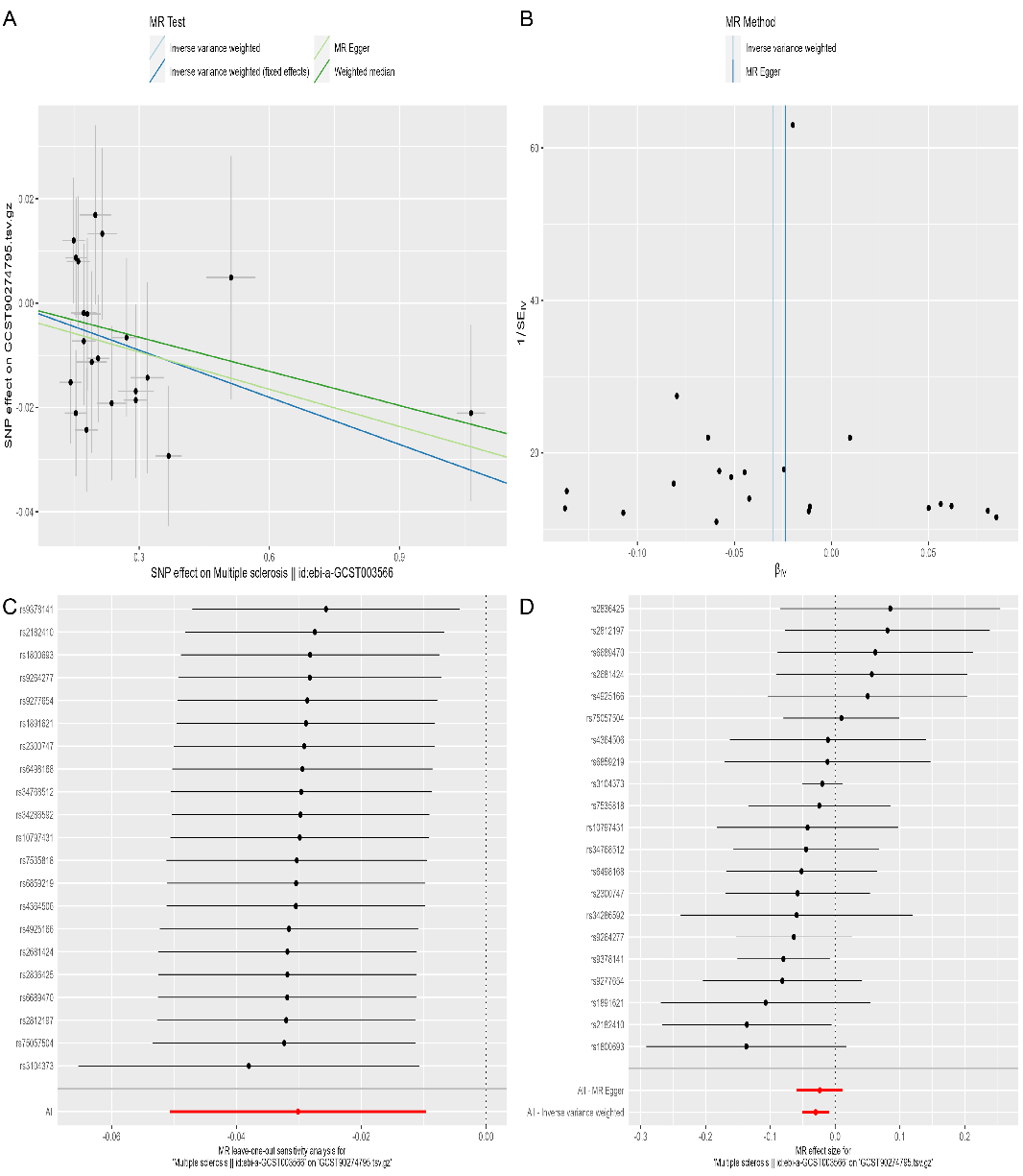
MR plots for the causal association of Multiple sclerosis on IL-10. A, Scatter plots. B, Funnel plot. C, Leave-one-out sensitivity analysis. D, Forest plot.

MR plots for the causal association of Hashimoto thyroiditis on acute rhinosinusitis. A, Scatter plots. B, Funnel plot. C, Leave-one-out sensitivity analysis. D, Forest plot.


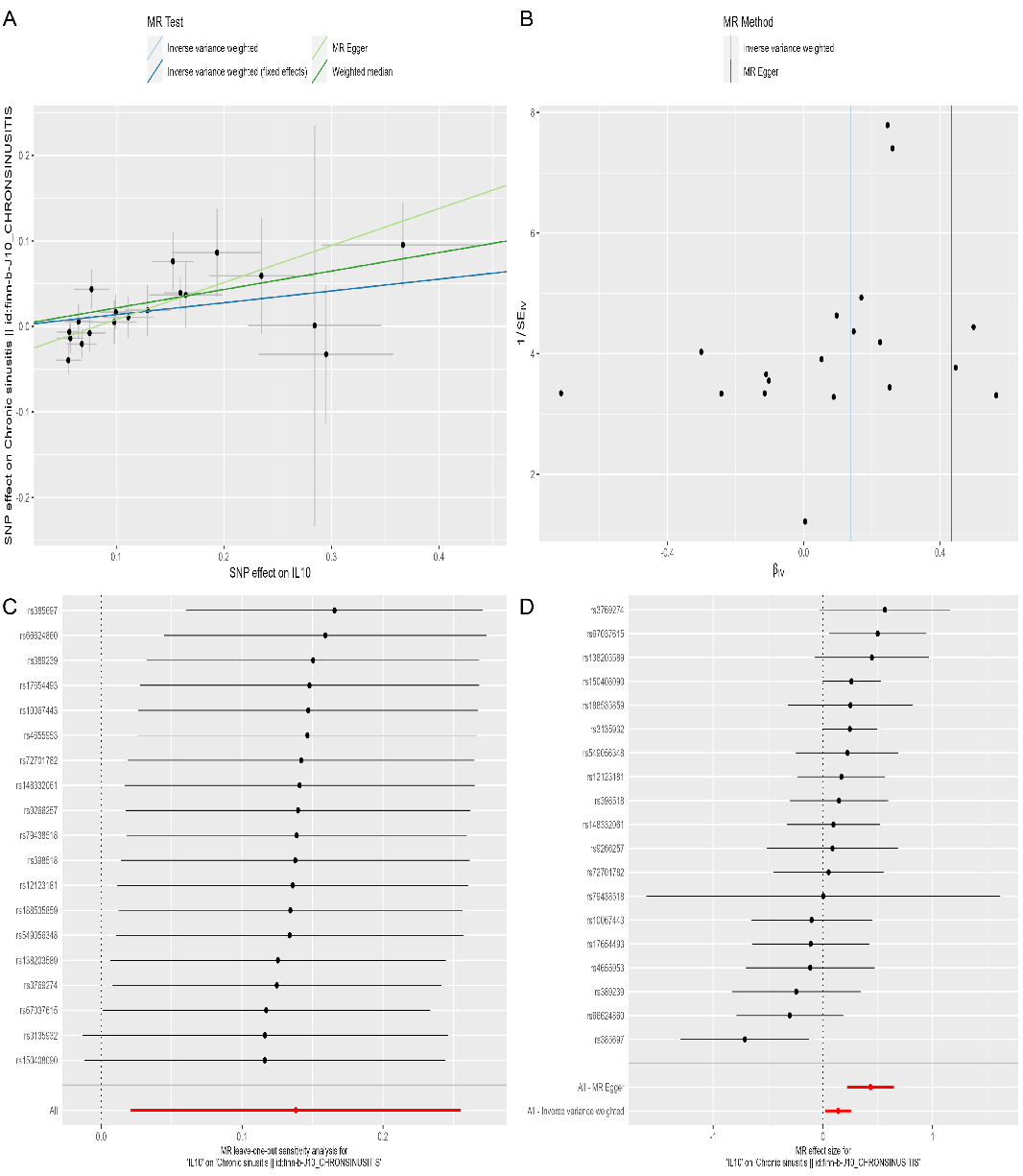
Supplementary Figure 27

MR plots for the causal association of IL-10 on chronic rhinosinusitis. A, Scatter plots. B, Funnel plot. C, Leave-one-out sensitivity analysis. D, Forest plot.


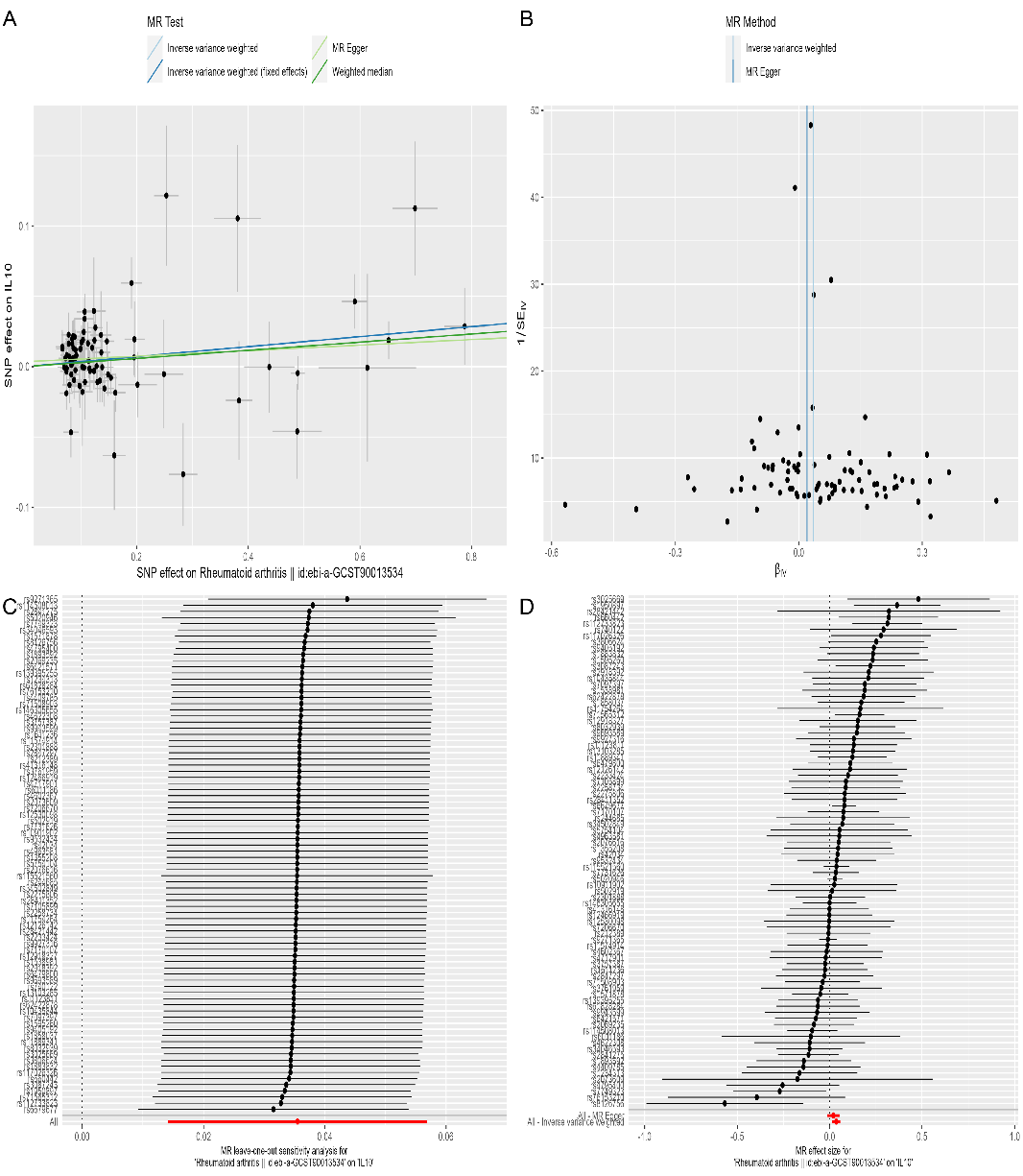
Supplementary Figure 28

MR plots for the causal association of Rheumatoid arthritis on IL-10. A, Scatter plots. B, Funnel plot. C, Leave-one-out sensitivity analysis. D, Forest plot.


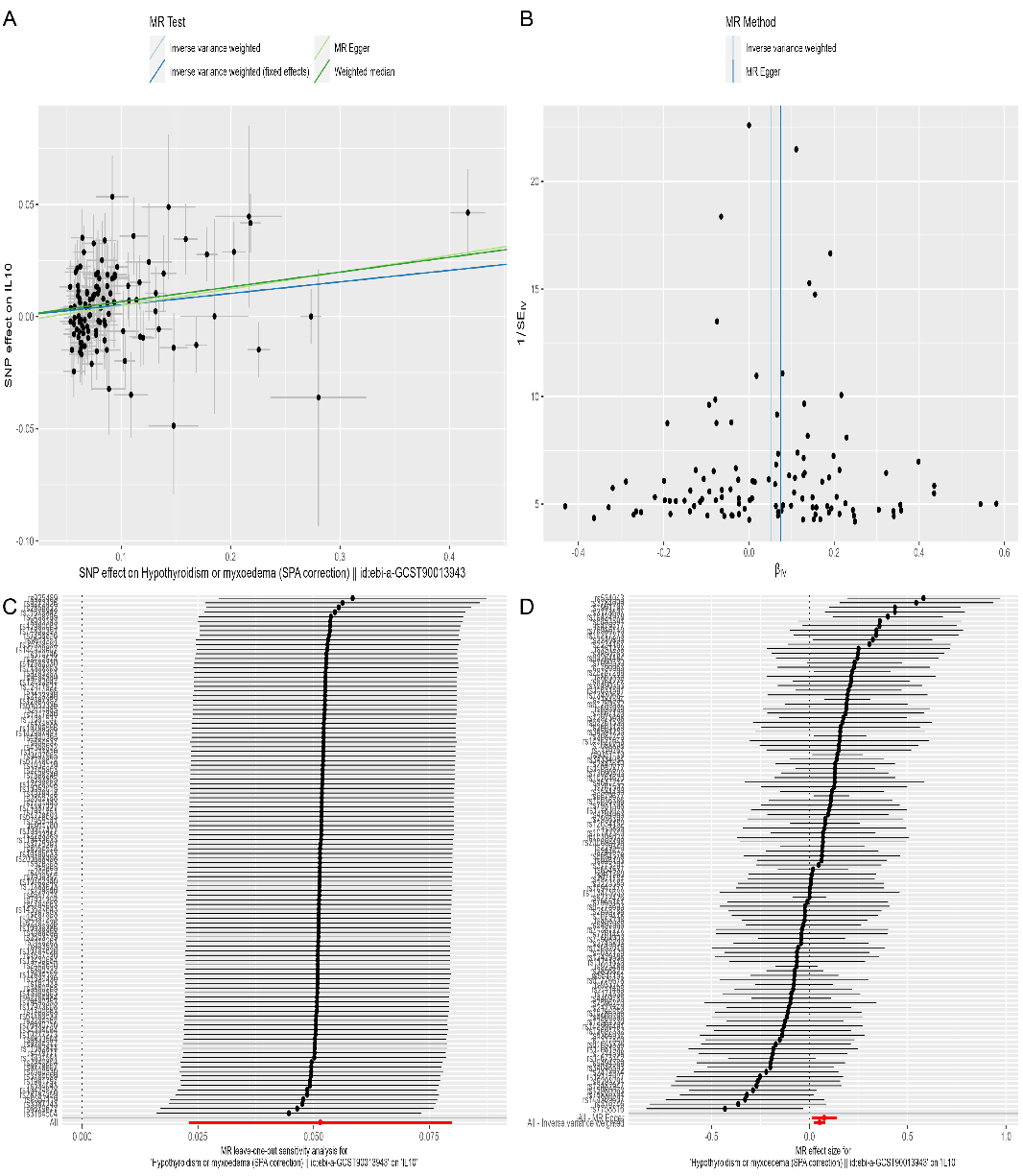
Supplementary Figure 29

MR plots for the causal association of Hypothyroidism or myxoedema on IL-10. A, Scatter plots. B, Funnel plot. C, Leave-one-out sensitivity analysis. D, Forest plot.


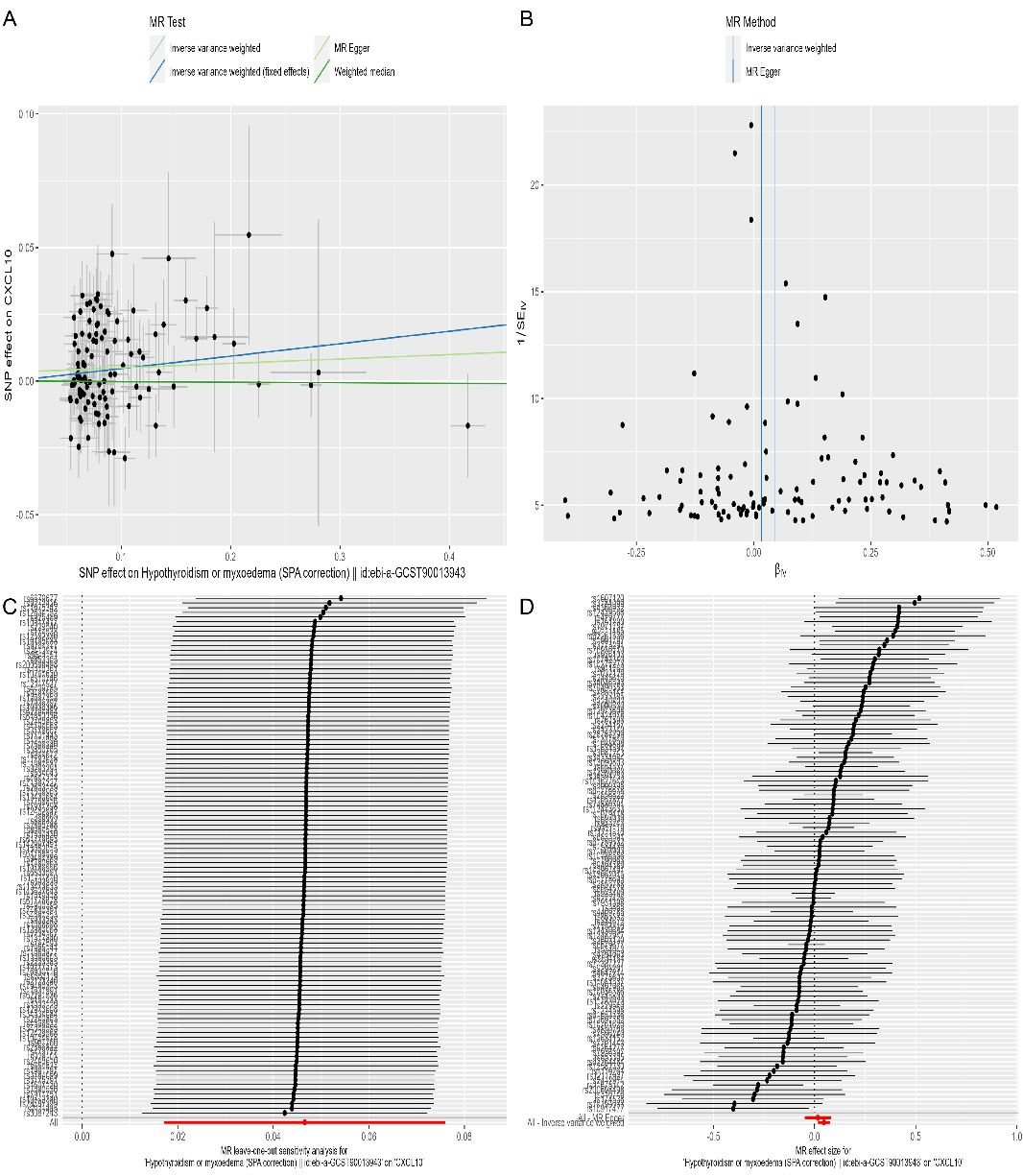
Supplementary Figure 30

MR plots for the causal association of Hypothyroidism or myxoedema on CXCL10. A, Scatter plots. B, Funnel plot. C, Leave-one-out sensitivity analysis. D, Forest plot.


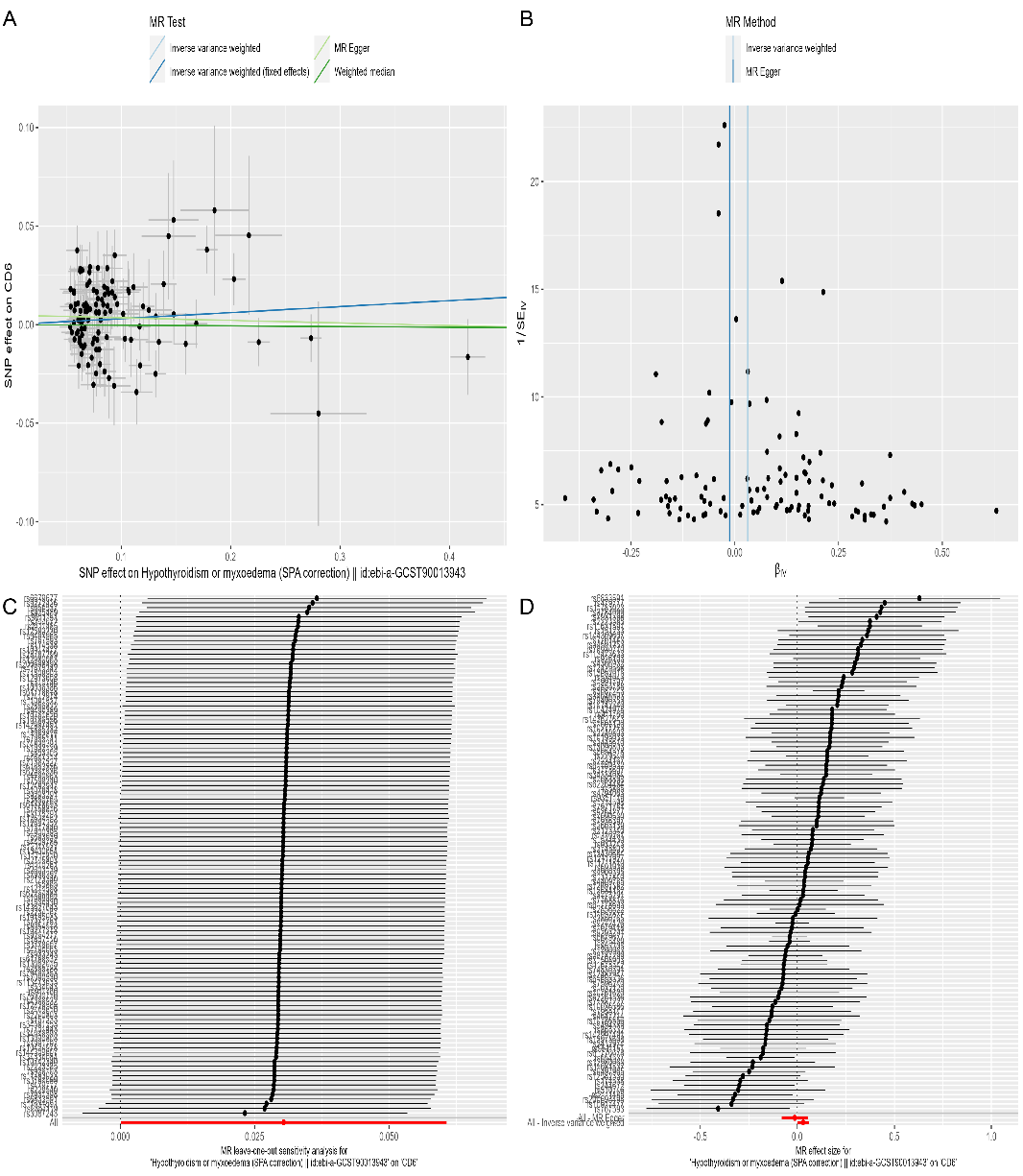
Supplementary Figure 31

MR plots for the causal association of Hypothyroidism or myxoedema on CD6. A, Scatter plots. B, Funnel plot. C, Leave-one-out sensitivity analysis. D, Forest plot.


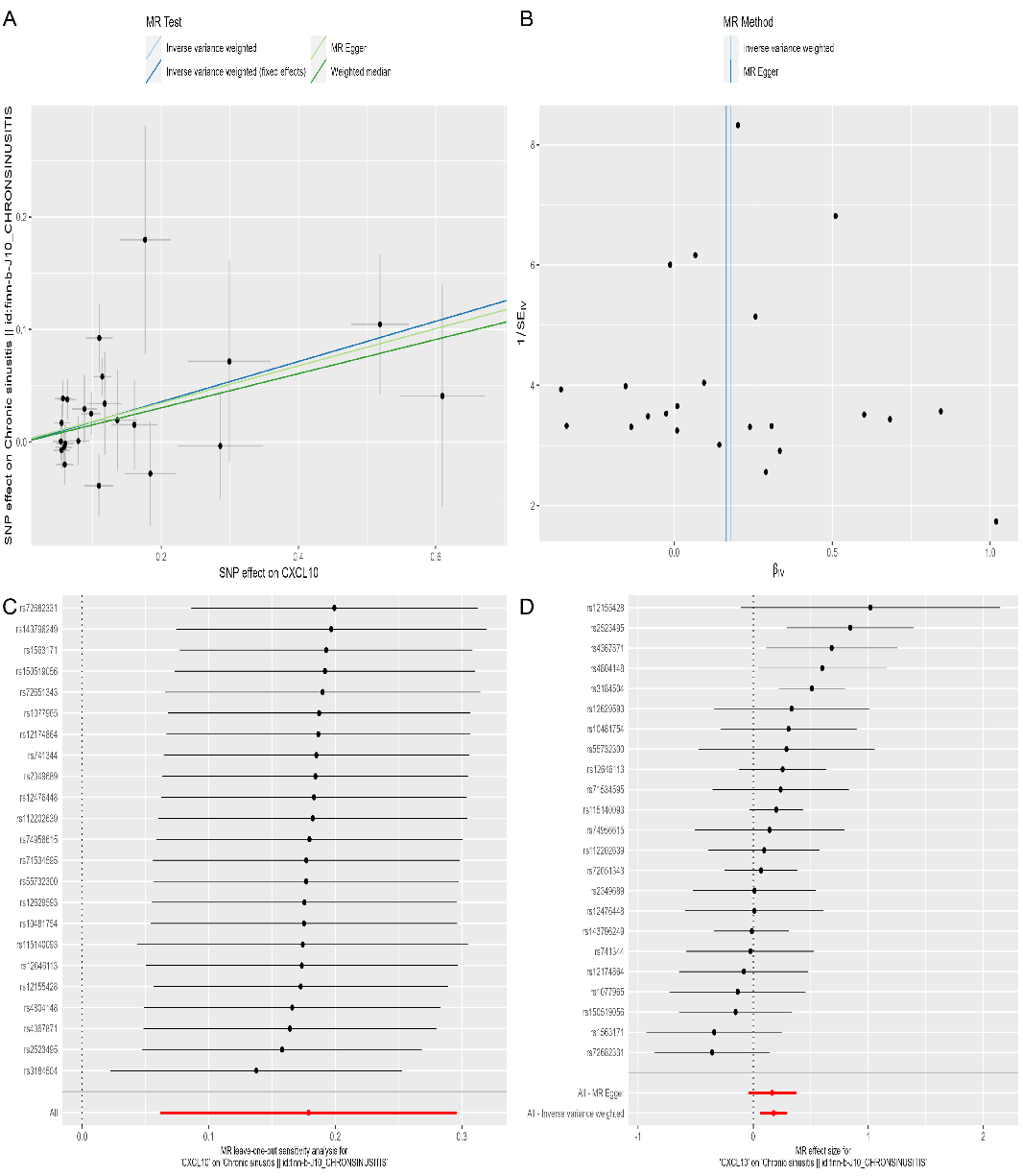
Supplementary Figure 32

MR plots for the causal association of CXCL10 on chronic rhinosinusitis. A, Scatter plots. B, Funnel plot. C, Leave-one-out sensitivity analysis. D, Forest plot.


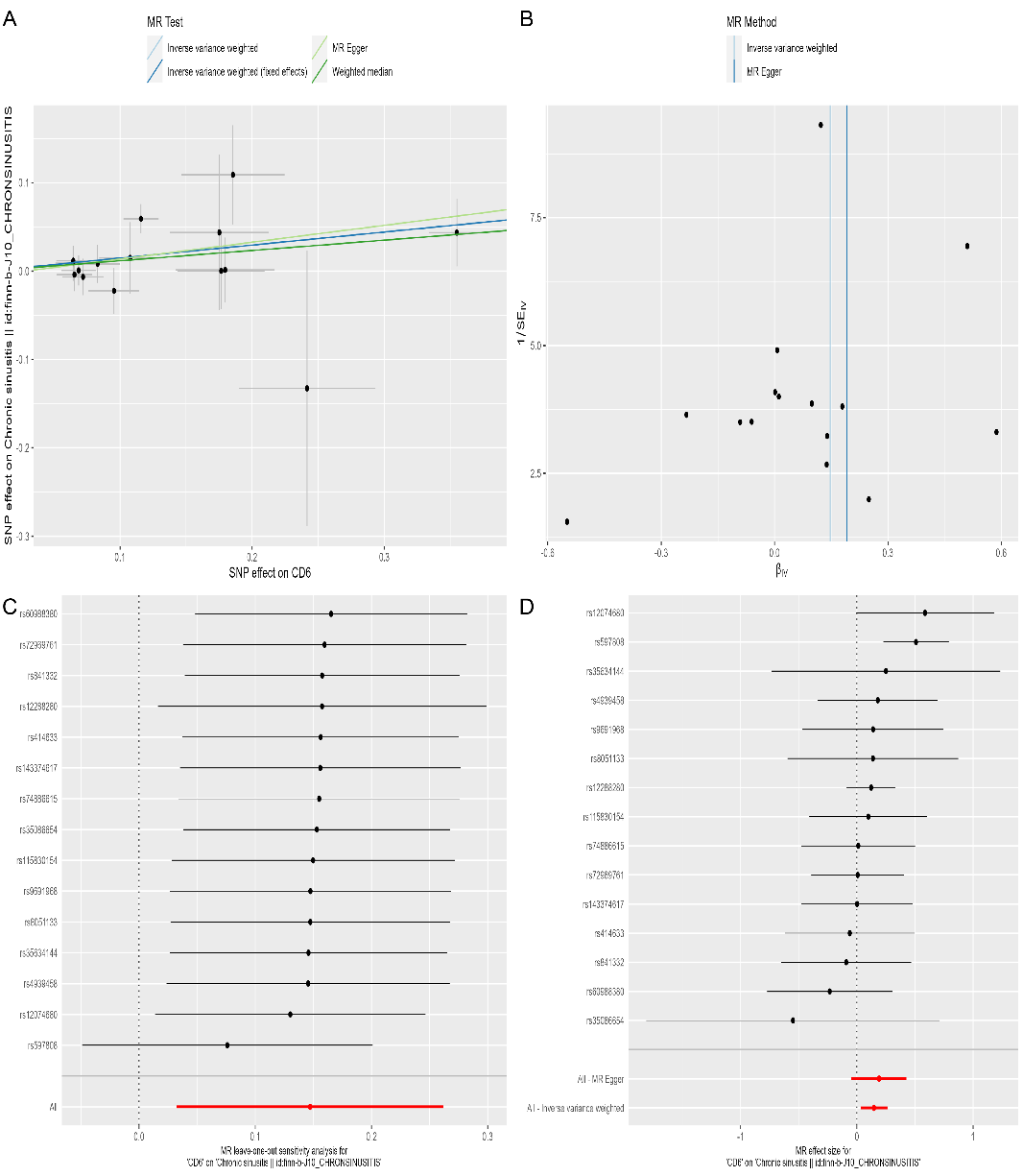
Supplementary Figure 33

MR plots for the causal association of CD6 on chronic rhinosinusitis. A, Scatter plots. B, Funnel plot. C, Leave-one-out sensitivity analysis. D, Forest plot.


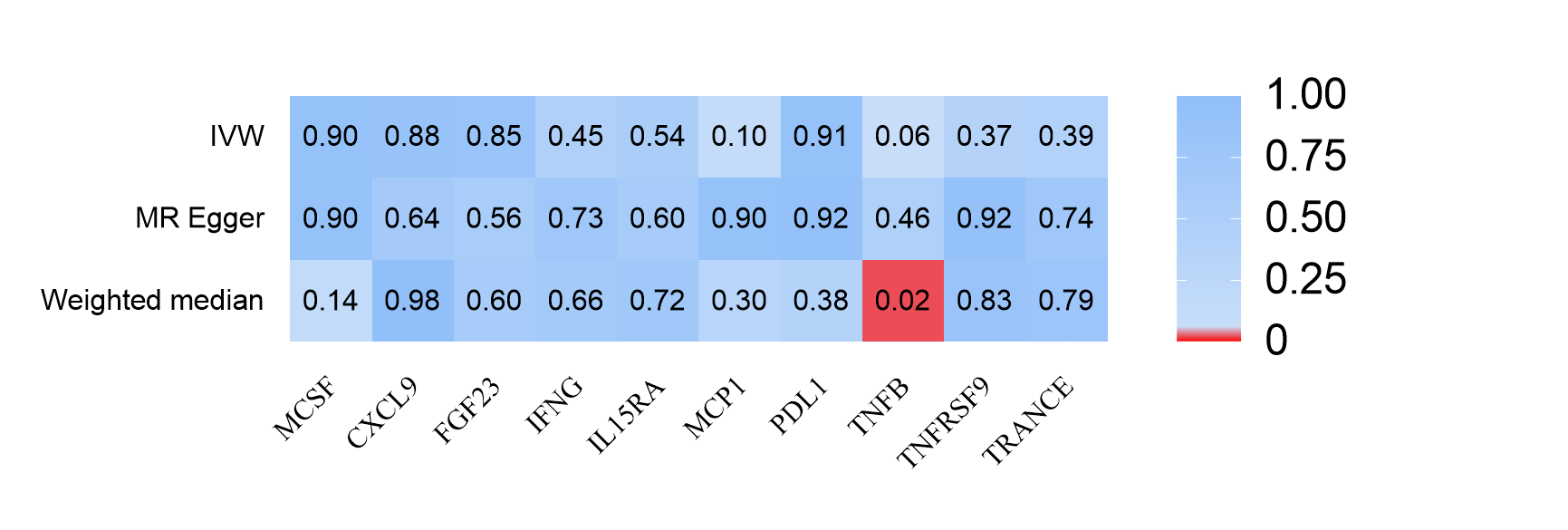
Supplementary Figure 34

The relationship between the inflammatory factors identified in the previous step and acute sinusitis was investigated using Mendelian randomization. The color gradient from blue to red indicates an increase in the significance of causation. IVW, inverse variance weighted.
